# A simple, ligand-free Cu(OH)_2_-catalyzed methoxylation of aryl halides

**DOI:** 10.1186/s13104-026-07720-x

**Published:** 2026-02-14

**Authors:** Karoline Nordli, Xavier Jouffroy, Meda Surdokaitė, Melina Flakowski, Anna Walter, Jørn H. Hansen

**Affiliations:** Department of Chemistry, Chemical Synthesis and Analysis Group, UiT The Arctic University of Norway, N-9037 Tromsø, Norway

**Keywords:** Methoxylation, Copper catalysis, Aryl halides, Ligand-free, Isotope labelling

## Abstract

**Objective:**

To develop a simple, rapid and cheap catalytic method for methoxylation of aryl halides for late-stage functionalization and demonstrate its utility in isotopic labelling.

**Results:**

A ligand-free, microwave-assisted method for methoxylation of aryl halides using copper(II) hydroxide as catalyst has been explored. The new method is simple and cheap, afforded synthetically useful yields in some cases, but does not seem to give generally higher yields in more substituted systems. The viability of isotopic labelling using the method has been demonstrated by ^13^C-incorporation at the methoxy group.

**Supplementary Information:**

The online version contains supplementary material available at 10.1186/s13104-026-07720-x.

## Introduction

The methoxy group (OMe) represents an important π-electron-donating aromatic substituent for property tuning in medicinal and organic materials chemistry [1, 2]. The group is prevalent in pharmaceuticals, natural products, agrochemicals, and organic materials. It is an important directing group, e.g. in electrophilic aromatic substitution (EAS) and ortho-functionalization chemistry, as well as a direct functionalization handle in Ni-catalysed methoxy-activation [3]. Moreover, it plays an important role in Positron Emission Tomography (PET) radiotracers because of the ease of C-11 radiolabelling of methoxy groups via bimolecular substitution [4]. The widespread utility and important role of this functional group have warranted numerous studies of direct methoxylation reactions [5–8].

The field of copper-catalyzed functionalization of aryl halides has been growing rapidly for the past decades, particularly in line with a stream of new developments in ligand designs and improved mechanistic understanding [9]. A number of review articles cover most of the literature in this area, including methoxylations of aryl halides with copper catalysts [5–8]. The predominant catalytic systems in the latter have been copper(I) salts with various ligand additives, and with generally long reaction times [5–9]. In light of this, we wanted to explore the possibility of using simple copper(II) salts without ligand additives and achieve rapid methoxylations of aryl halides under microwave irradiation. We further envisioned the use of the reaction in isotopic labelling reactions for medical imaging applications.

In this research note, we describe the exploration of a simple and cheap approach to methoxylation of aryl halides using copper(II) hydroxide as catalyst. We present the current substrate scope and demonstrate isotopic labelling of the methoxy group using ^13^C-labelled methanol.

## Results and discussion

We have pursued the development of a cheap, fast and procedurally convenient methodology for methoxylation at aryl halides to apply this to late-stage functionalization and isotopic labelling. Based on early works, simple copper-salts can be effective in catalyzing the direct methoxylation of aryl halides [10]. Moreover, a recent study by Xiao et al. [11] suggested to us that the addition of an additional copper ligand could be omitted if the reaction was conducted in neat methanol, which could also be beneficial for the reaction by providing a large excess of methoxy groups. Although reported with copper(I)-salts, we envisioned that more stable, simple copper(II)-salts could be employed, and we chose Cu(OH)_2_ as a starting point based on the work of Xiao et al. [11].

**Fig. 1:**
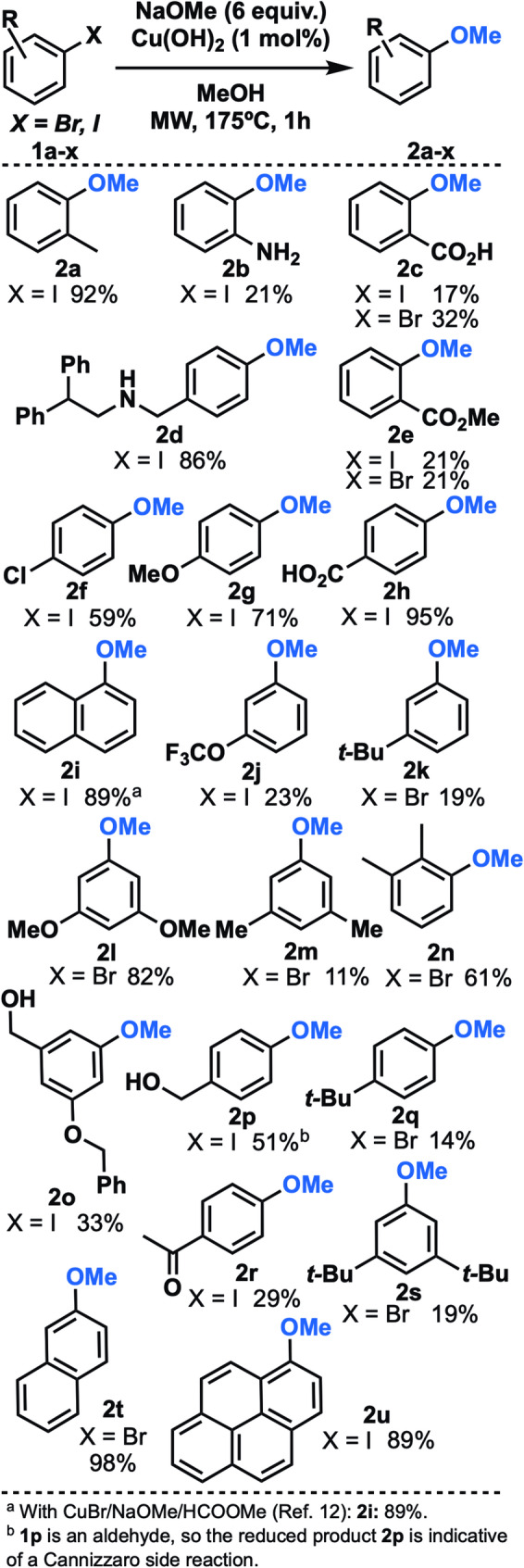
Cu-catalyzed methoxylations

An initial screening demonstrated that microwave heating is advantageous (Table S1) and that low catalyst loading (1 mol%) is possible without extra ligands added (Table S2). A survey of differentially substituted aryl iodides and bromides **1a-u** resulted in products **2a-u** summarized in Fig. 1. Generally, mono-, di- and trisubstituted systems are tolerated and also *ortho-*, *meta*- and *para*- substitution patterns. The chemical yields are low in the 10–30% range for about half the examples and there is not a clear trend in electronic or steric effects of the high-yielding reactions. The more electron-neutral aryl systems give synthetically useful yields of **2a**,**2d**,**2i**,**2t** and **2u** in the reaction, ranging from 69 to 98%, whereas more electron-rich or poor systems give a wider range of yields. The *para*-formyl substituted aryl iodide **1p** gave 51% yield of **2p**. The Cannizzaro reaction has occurred as a side reaction and the corresponding benzylic alcohol was the only isolated product. Furthermore, there were no noticeable significant differences in yields between aryl iodides and aryl bromides. The reaction with 1-iodonaphthalene **1i** was also repeated with another set of conditions reported earlier in which CuBr was employed as catalyst with methyl formate as co-catalyst [12], and both methods gave the same results.

**Fig. 2:**
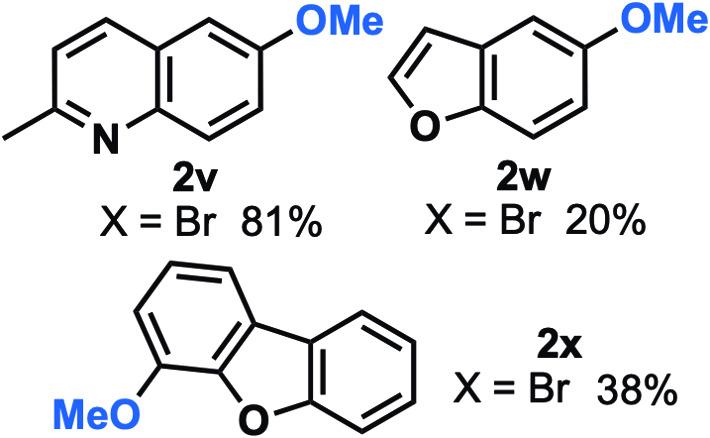
Methoxylation of heteroaryl bromides

Late-stage methoxylation of heterocycles is of utmost interest for the development of medicinal agents and organic materials [1, 2]. We surveyed a few systems to generate products **2v-x** (Fig. 2). A high yield of 81% was observed for the 2-methylquinoline-system, which is consistent with higher observed yields for relatively electron-neutral systems in Fig. 1. Decent yields were also obtained in the heterocyclic benzofuran systems to give products **2w** and **2x** in 20% and 38% yields, respectively. This demonstrates compatibility with some heterocyclic motifs.

**Fig. 3:**
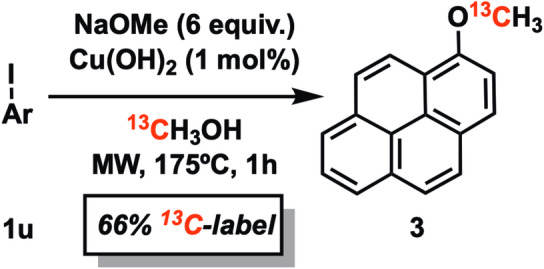
^13^C-methoxylation of 1-iodopyrene

An interesting area of potential application of the methoxylation reaction would be in isotopic labelling, e.g. C-11 labelling of bioactives for PET-imaging [4]. In lieu of accessible C-11, we decided to demonstrate the viability of isotopic labelling by using C-13 labelled methanol. Indeed, conducting the reaction of 1-iodopyrene in C-13 methanol afforded 66% incorporation of the C-13 label in the resulting 1-methoxypyrene **3** (Fig. 3). The remaining C-12 labelled product must come from sodium methoxide. It may be possible to increase the amount of labelled product by equilibrating the NaOMe/[^13^C]MeOH mixture longer in advance of the reaction.

In summary, we have conducted a survey of conditions and the substrate scope of a copper(II) hydroxide catalyzed methoxylation reaction employing sodium methoxide in methanol in a microwave-assisted reaction. The reaction is operationally simple, employs low catalyst loading and does not require addition of ligands for the copper-catalyst. The chemical yields are variable, but synthetically useful yields could be obtained in many instances representing various substitution patterns including heterocyclic substrates. Notably, the methodology can be used for isotopic labelling at the methoxy group by employing isotopically enriched methanol as the reaction medium.

## Methods

Unless otherwise noted, purchased chemicals were used as received without further purification. Sodium methoxide was purchased from Merck 156,256 -25ML Sodium methoxide solution, 25 wt% in methanol and was used without purification or analysis Solvents were dried according to standard procedures [13] over molecular sieves and degassed with argon for at least 5 min. Microwave reactions were conducted in a Monowave 300 by Anton Paar. Flash chromatography was carried out on silica gel 60 (230–400 mesh). Thin layer chromatography was carried out using TLC Silica Gel 60 F254 (Merck) and visualized by short-wavelength ultraviolet light or by treatment with an appropriate stain. High-resolution mass spectra HRMS(ESI) were recorded from methanol solutions on a LTQ Orbitrap XL (Thermo Scientific) in either positive or negative electrospray ionization (ESI) mode. NMR spectra were obtained on a 400 MHz Bruker Avance III HD at 20 °C. The shifts are reported in ppm relative to the solvent residual peak (CDCl_3_: δH 7.26 and δC 77.16; Methanol-d4: δH 3.31 and δC 49.00; DMSO-d6: δH 2.51 and δC 39.52). ^13^C-NMR spectra were obtained with ^1^H decoupling. Data is presented as follows: chemical shift, multiplicity (s = singlet, bs = broad singlet, d = doublet, t = triplet, q = quartet, dt = doublet of triplets, m = multiplet), coupling constant (J in Hz). FT-IR spectra were recorded on a Cary 630 FTIR (Agilent Technologies). GC-MS analysis was performed on a TRACE GC ULTRA, ITQ 1100 instrument with a SUPELCO analytical SLB™-5ms Fused Silica Capillary Column 30 m x 0.2 μm film thickness.

## Limitations

The chemical yields were variable, and it is difficult to discern a clear structure-reactivity relationship. The problem of product volatility is likely a contributing factor. The tolerance towards different electron-withdrawing and donating groups is ok, but the yields are low to moderate. This may also be in part due to instability during the the high reaction temperature during the 1-hour microwave reaction conditions. For the heterocycles, product extractability seemed to pose a problem in certain cases. The major byproducts generally observed were hydroxylation [14] if too much water was present, or dehalogenation of the aryl halide which to some extent occurs thermally. The method does not appear to offer major synthetic advantages over other published methods other than price and practicability of the procedure.

## Supplementary Information

Below is the link to the electronic supplementary material.

Supplementary Material 1.

## Data Availability

Spectroscopic data for all final products can be found in a separate supplementary file and is also available from the corresponding author upon request.

## Abbreviations

Me: Methyl
equiv.: Equivalents
EAS: Electrophilic aromatic substitution
MW: Microwave
PET: Positron emission tomography
HRMS: High-resolution mass spectrometry
rt: Room temperature
TLC: Thin-layer chromatography
NMR: Nuclear magnetic resonance
FT-IR: Fourier transform-infrared spectroscopy
