# Supplementary material for: A simple, ligand-free Cu(OH)2-catalyzed methoxylation of aryl halides
Source: BMC Res Notes. 2026 Feb 14;19:122. doi: 10.1186/s13104-026-07720-x (PMC13011408; doi:10.1186/s13104-026-07720-x)
Supplement: Supplementary file 1 — Supplementary Material 1. [file 13104_2026_7720_MOESM1_ESM.pdf]

Supporting Information for

A simple, ligand-free $Cu(OH)_2$-catalyzed methoxylation of aryl halides

*Karoline Nordli, Xavier Jouffroy, Meda Surdokaitė, Melina Flakowski, Anna Walter, and Jørn H. Hansen**

Department of Chemistry, UiT The Arctic University of Norway, Chemical Synthesis and Analysis Group, N-9037 Tromsø, Norway

** Corresponding author e-mail: jorn.h.hansen@uit.no*

Table of contents

1. Initial studies 2
2. Screening of reaction conditions 2
3. Experimental section 3
   3.1 General considerations 3
   3.2 General procedure for $Cu(OH)_2$-catalyzed synthesis of functionalized anisoles 3
4. Characterization 5
5. Spectral information 13
6. References 38

1. Initial studies

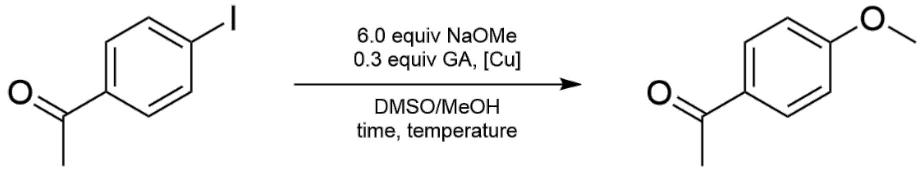

Table S1. Initial studies

Entry	Time	Temperature (°C)	Solvent DMSO:MeOH	Cu source	Heating	Yield (%)
1	6 h	120	1:1	$Cu(OH)_2$	Conventional	15
2	24 h	120	1:1	$Cu(OH)_2$	Conventional	28
3	20 h	120	1:1	CuCl	Conventional	23
4	**15 min**	**160**	**Only MeOH**	**$Cu(OH)_2$**	**Microwave**	29

GA = Glycolic acid

2. Screening of reaction conditions

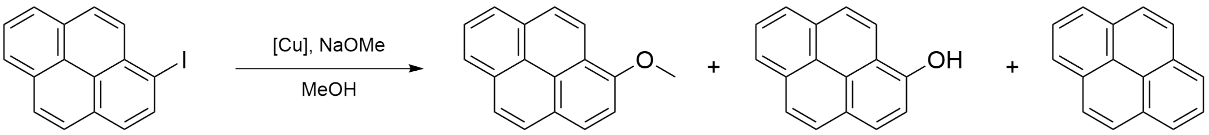

1a **2a** **3a** **4a**

Table S2. Screening conditions for copper-catalyzed methoxylation of iodopyrene.

Entry	Cu-source (mol%)	NaOMe (eq.)	Glycolic acid (eq.)	Temp (°C)	Time (min)	Conversion (%)	Relative yield (%, LC-UV) **2a**	Relative yield (%, LC-UV) **3a**	Relative yield (%, LC-UV) **4a**
1	$Cu(OH)_2$ (5%)	6	0,3	160	15	-	85	-	-
2	$Cu(OH)_2$ (5%)	6	0,3	160	15	-	89	-	-
3	$Cu(OH)_2$ (5%)	6	0,3	160	5	78	69	4	6
4	$Cu(OH)_2$ (5%)	6	0,3	160	10	100	5	0	95
5	$Cu(OH)_2$ (5%)	6	0,3	160	10	96	86	5	6
6	$Cu(OH)_2$ (5%)	6	0,3	120	15	10	9	0	1
7	$Cu(OH)_2$ (5%)	2	0,3	160	15	100	70	2	28
8	$Cu(OH)_2$ (5%)	6	0,1	160	15	100	85	6	9
9	$Cu(OH)_2$ (1%)	6	0,3	160	15	100	89	6	4
10	$Cu(OH)_2$ (1%)	6	0	160	15	100	85	4	11
11	$Cu(OAc)_2$ (5%)	6	0	160	15	100	79	5	16
12	Cu(I) (5%)	6	0	160	15	98	77	5	16
13	$Cu(Cl)_2$ (5%)	6	0	160	15	99	76	4	19
14	No catalyst	6	0	160	15	40	2	0	37
15	$Cu(OH)_2$ (1%)	6	0	160	15	100	85	4	11
16[a]	$Cu(OH)_2$ (1%)	6	0	160	15	100	70	10	19

[a] *added 4 equiv. of water.*

3. Experimental section

3.1 General considerations

Unless otherwise noted, purchased chemicals were used as received without further purification. Sodium methoxide was purchased from Merck 156256 -25ML Sodium methoxide solution, 25 wt% in methanol and was used without purification or analysis (Wethman et al., 2021). Solvents were dried according to standard procedures over molecular sieves and degassed with argon for at least 5 min. Microwave reactions were conducted in a Monowave 300 by Anton Paar. Flash chromatography was carried out on silica gel 60 (230-400 mesh). Thin layer chromatography was carried out using TLC Silica Gel 60 F254 (Merck) and visualized by short-wavelength ultraviolet light or by treatment with an appropriate stain. High-resolution mass spectra HRMS(ESI) were recorded from methanol solutions on a LTQ Orbitrap XL (Thermo Scientific) in either positive or negative electrospray ionization (ESI) mode. NMR spectra were obtained on a 400 MHz Bruker Avance III HD at 20 °C. The shifts are reported in ppm relative to the solvent residual peak ($CDCl_3$: δH 7.26 and δC 77.16). ^{13}C-NMR spectra were obtained with ^{1}H decoupling. Data is presented as follows: chemical shift, multiplicity (s = singlet, bs = broad singlet, d = doublet, t = triplet, q = quartet, dt = doublet of triplets, m = multiplet), coupling constant (J in Hz). FT-IR spectra were recorded on a Cary 630 FTIR (Agilent Technologies).

3.2 General procedure for $Cu(OH)_2$-catalyzed synthesis of functionalized anisoles

A microwave reactor was charged with aryl halide (0.5 mmol, 1.0 equiv.), $Cu(OH)_2$ (0.005 mmol, 0.01 equiv.), NaOMe (3.0 mmol, 6.0 equiv.), and MeOH (3.0 mL/mmol) under argon using Schlenk line technique. The mixture was stirred at 175 °C for 60 min in the microwave. The reaction mixture was allowed to cool to room temperature, poured into water, and then adjusted to pH = 8-9 with 2 N HCl. The aqueous phase was extracted twice with diethyl ether, and the combined organic layers were washed with H_2O and brine, dried over anhydrous Na_2SO_4, and concentrated under vacuum. Purification of the crude product by column chromatography (diethyl ether/pentane) afforded the functionalized anisoles.

See Figures S1 and S2 for an example of a microwave run and experimental settings. The microwave was set to provide maximum power (Figure S1, blue line) until the set temperature was reached, and then keep the temperature constant.

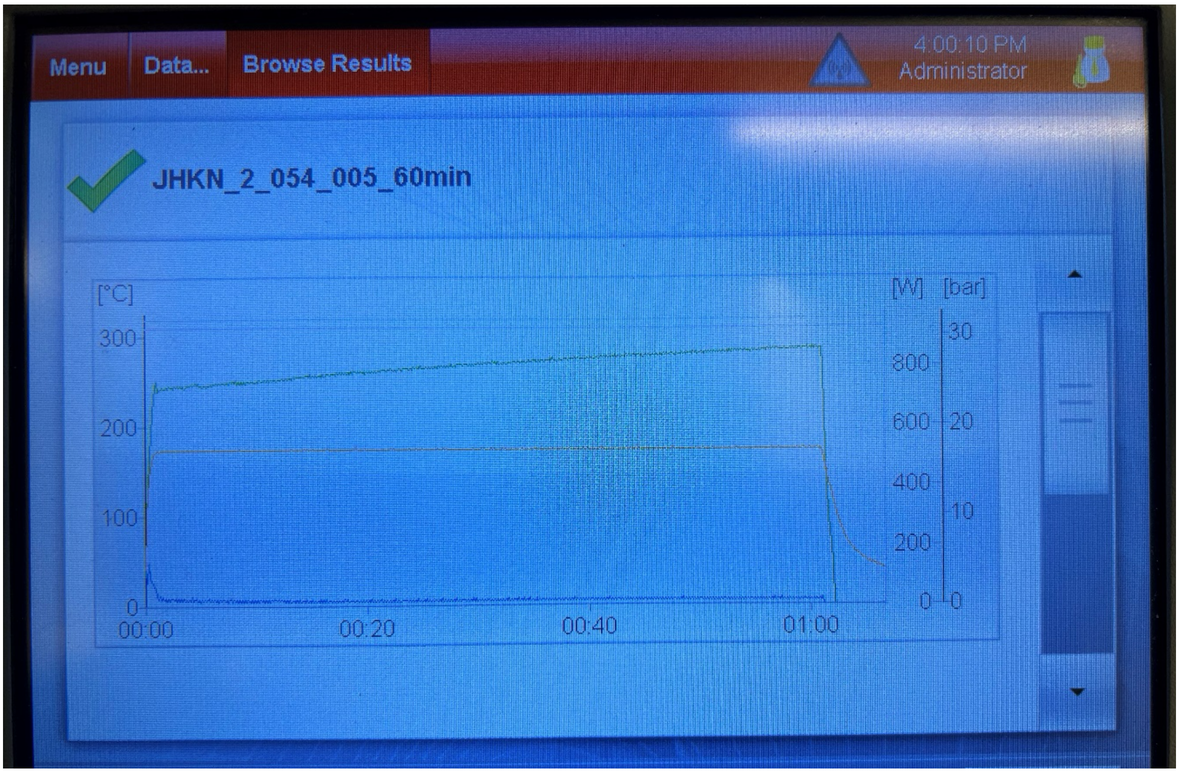

Figure S1. A typical microwave run profile showing power (blue), temperature (yellow) and pressure (green) in the reactor.

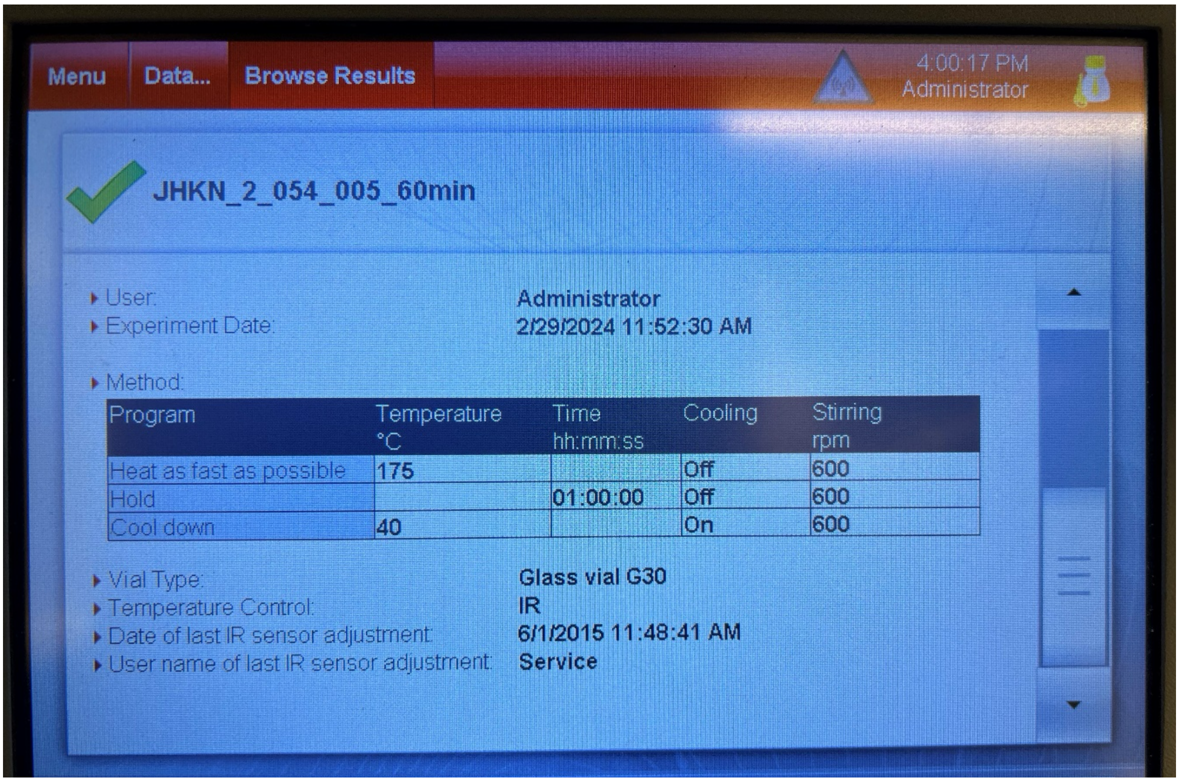

Figure S2. Typical microwave program.

4. Characterization

2a

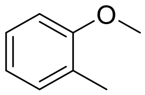

Prepared according to the general procedure. The crude product was purified by silica gel column chromatography (pentane:diethyl ether, 100:2) to obtain the desired product as a clear oil (56 mg, 92 %). R_f = 0,87 (pentane:diethyl ether, 100:2). ^{1}**H NMR** (400 MHz, $CDCl_3$) δ 7.26 – 7.15 (m, 2H), 6.97 – 6.85 (m, 2H), 3.88 (s, 3H), 2.29 (s, 3H) ppm. ^{13}**C NMR** (101 MHz, $CDCl_3$) δ 157.8, 130.7, 126.9, 126.7, 120.3, 109.9, 55.3, 16.3 ppm. The data is consistent with literature (Haydl & Hartwig, 2019).

2b

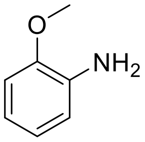

Prepared according to the general procedure. The crude product was purified by silica gel column chromatography (pentane:diethyl ether, 8:1) to obtain the desired product as an orange oil (14 mg, 21 %). R_f = 0,36 (pentane:diethyl ether, 8:1). ^{1}**H NMR** (400 MHz, $CDCl_3$) δ 6.83 – 6.77 (m, 2H), 6.76 – 6.71 (m, 2H), 3.85 (s, 3H) ppm. ^{13}**C NMR** (101 MHz, $CDCl_3$) δ 149.0, 122.8, 119.6, 115.9, 110.6, 55.6 ppm. The data is consistent with literature.(Panda et al., 2023)

2c

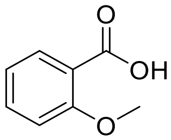

Prepared according to the general procedure. The crude product was purified by silica gel column chromatography (pentane:diethyl ether, 100:20 → 100:40 → 100:50) to obtain the desired product (24 mg, 32 %). ^{1}**H NMR** (400 MHz, $CDCl_3$) δ 10.80 (s, 1H), 8.19 (dd, *J* = 7.8, 1.8 Hz, 1H), 7.62 – 7.51 (m, 1H), 7.14 (td, *J* = 7.6, 1.0 Hz, 1H), 7.06 (d, *J* = 8.4 Hz, 1H), 4.08 (s, 3H). ^{13}**C NMR** (101 MHz, $CDCl_3$) δ 165.6, 158.2, 135.2, 134.0, 122.4, 117.7, 111.8, 56.8. The data is consistent with literature.(Xu et al., 2023)

2d

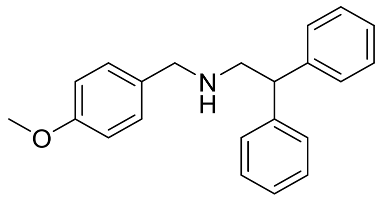

Prepared according to the general procedure. The crude product was purified by silica gel column chromatography (pentane:EtOAc, 5:5) to obtain the desired product as a clear oil (130 mg, 86 %). R_f = 0,34 (pentane:EtOAc, 5:5). **^{1}H NMR** (400 MHz, $CDCl_3$) δ 7.31 – 7.08 (m, 12H), 6.84 – 6.76 (m, 2H), 4.20 (t, *J* = 7.6 Hz, 1H), 4.09 (q, *J* = 7.1 Hz, 1H), 3.73 (s, 3H), 3.19 (d, *J* = 7.7 Hz, 2H) ppm. **^{13}C NMR** (101 MHz, $CDCl_3$) δ 171.1, 158.6, 143.0, 132.3, 129.3, 128.6, 128.1, 127.7, 126.6, 125.1, 119.2, 114.5, 57.0, 50.3, 41.7 ppm. The data is consistent with literature.(Collins, 2004)

2e

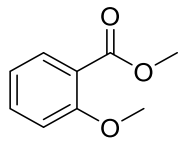

Prepared according to the general procedure. The crude product was purified by silica gel column chromatography (pentane:diethyl ether, 100:10 → 100:50) to obtain the desired product as a clear oil (18 mg, 21 %). **^{1}H NMR** (400 MHz, $CDCl_3$) δ 7.79 (dd, *J* = 7.8, 1.8 Hz, 1H), 7.51 – 7.42 (m, 1H), 7.02 – 6.94 (m, 2H), 3.91 (s, 3H), 3.89 (s, 3H). **^{13}C NMR** (101 MHz, $CDCl_3$) δ 166.9, 159.3, 133.7, 131.8, 120.3, 120.2, 112.1, 56.1, 52.2. The data is consistent with literature.(Bai et al., 2012)

2f

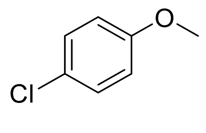

Prepared according to the general procedure. The crude product was purified by silica gel column chromatography (pentane:diethyl ether, 100:5) to obtain the desired product (42 mg, 59 %). **^{1}H NMR** (400 MHz, $CDCl_3$) δ 7.24 (d, *J* = 8.5 Hz, 2H), 6.83 (dd, *J* = 8.6, 1.5 Hz, 2H), 3.79 (s, 3H). The data is consistent with literature.(Liang et al., 2019)

2g

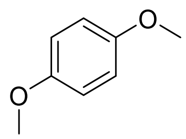

Prepared according to the general procedure. The crude product was purified by silica gel column chromatography (pentane:diethyl ether, 100:0 → 100:5) to obtain the desired product as a white solid (49 mg, 71 %). ^{1}**H NMR** (400 MHz, $CDCl_3$) δ 6.85 (s, 4H), 3.77 (s, 6H). ^{13}**C NMR** (101 MHz, $CDCl_3$) δ 154.0, 114.8, 55.9. The data is consistent with literature.(Zhou et al., 2022)

2h

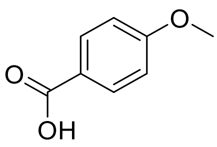

Prepared according to the general procedure. The crude product was purified by silica gel column chromatography (chloroform:methanol, 9:1) to obtain the desired product as a white solid (65 mg, 95 %). R_f = 0,51 (chloroform:methanol, 9:1). ^{1}**H NMR** (400 MHz, $CDCl_3$) δ 8.07 (d, *J* = 8.5 Hz, 2H), 6.95 (d, *J* = 8.3 Hz, 2H), 3.88 (s, 3H) ppm. The data is consistent with literature.(Meng et al., 2017)

2i

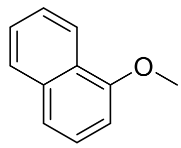

Prepared according to the general procedure. The crude product was purified by silica gel column chromatography (pentane:diethyl ether, 100:0 → 100:5) to obtain the desired product as a yellow oil (70 mg, 89 %). ^{1}**H NMR** (400 MHz, $CDCl_3$) δ 8.33 – 8.24 (m, 1H), 7.82 (dt, *J* = 7.2, 3.0 Hz, 1H), 7.58 – 7.35 (m, 4H), 6.84 (dd, *J* = 7.4, 1.4 Hz, 1H), 4.02 (s, 3H). ^{13}**C NMR** (101 MHz, $CDCl_3$) δ 155.6, 134.6, 127.6, 126.5, 126.0, 125.7, 125.3, 122.1, 120.4, 103.9, 55.6. The data is consistent with literature.(Kawabata & Matsubara, 2023)

2j

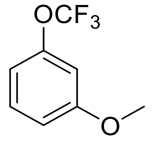

Prepared according to the general procedure. The crude product was purified by silica gel column chromatography (pentane:diethyl ether, 4:1) to obtain the desired product as a yellow oil (44 mg, 23 %). **^{1}H NMR** (400 MHz, $CDCl_3$): δ 7.28 (m, 1H), 6.82 (m, 2H), 6.76 (s, 1H), 3.82 (s, 3H). **^{13}C NMR** (100 MHz, $CDCl_3$): δ 156.6, 150.2, 150.2, 130.4, 120.4 (q, $^1J_{CF}$ = 258 Hz), 113.8, 113.1, 108.5, 31.0. The data is consistent with literature. (Huang et al., 2011).

2k

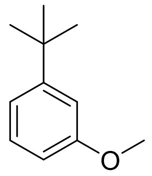

Prepared according to the general procedure. The crude product was purified by silica gel column chromatography (pentane:diethyl ether, 4:1) to obtain the desired product as a clear oil (31 mg, 19 %). **^{1}H NMR** (400 MHz, $CDCl_3$) δ 7.28 – 7.20 (m, 1H), 7.03 – 6.98 (m, 1H), 6.96 (t, *J* = 2.1 Hz, 1H), 6.73 (dd, *J* = 8.2, 2.5 Hz, 1H), 3.82 (s, 3H), 1.32 (s, 9H). **^{13}C NMR** (101 MHz, $CDCl_3$) δ 159.4, 153.0, 129.0, 117.9, 112.03, 110.0, 55.2, 34.8, 31.3(3C). The data is consistent with literature.(Bhadra et al., 2013)

2l

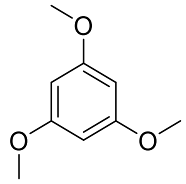

Prepared according to the general procedure. The crude product was purified by silica gel column chromatography (pentane:diethyl ether, 100:0 → 100:10) to obtain the desired product as a white solid (69 mg, 82 %). **^{1}H NMR** (400 MHz, $CDCl_3$) δ 6.09 (s, 3H), 3.77 (s, 9H). **^{13}C NMR** (101 MHz, $CDCl_3$) δ 161.7, 93.1, 55.4. The data is consistent with literature. (Sum et al., 2018)

2m

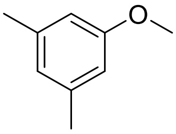

Prepared according to the general procedure. The crude product was purified by silica gel column chromatography (pentane:diethyl ether, 10:1) to obtain the desired product as a clear oil (15 mg, 11 %). **^{1}H NMR** (400 MHz, $CDCl_3$): δ 6.6 (s, 1H), 6.2 (s, 2H), 3.78 (s, 3H), 2.3 (s, 6H). **^{13}C NMR** (101 MHz, CDCl3): δ 159.6, 139.2, 122.5, 111.7, 55.1, 21.4. This data is consistent with literature.(Fan et al., 2018)

2n

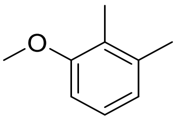

Prepared according to the general procedure. The crude product was purified by silica gel column chromatography (pentane) to obtain the desired product as a clear oil (42 mg, 61 %). R_f = 0,52 (pentane). **^{1}H NMR** (400 MHz, $CDCl_3$) δ 7.11 (t, J = 7.9 Hz, 1H), 6.83 (d, J = 7.6 Hz, 1H), 6.76 (d, J = 8.2 Hz, 1H), 3.86 (s, 3H), 2.32 (s, 3H), 2.20 (s, 3H) ppm. **^{13}C NMR** (101 MHz, $CDCl_3$) δ 157.6, 137.9, 125.8, 125.1, 122.3, 107.9, 55.6, 20.1, 11.6 ppm. The data is consistent with literature.(Eskildsen et al., 2000)

2o

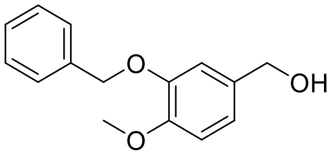

Prepared according to the general procedure. The crude product was purified by silica gel column chromatography (pentane:diethyl ether, 6:4) to obtain the desired product as a white solid (40 mg, 33 %). R_f = 0,48 (pentane:diethyl ether, 6:4). **^{1}H NMR** (400 MHz, $CDCl_3$) δ 7.45 (d, J = 6.7 Hz, 2H), 7.37 (t, J = 7.3 Hz, 2H), 7.30 (t, J = 7.3 Hz, 1H), 6.95 (s, 1H), 6.92 – 6.85 (m, 2H), 5.15 (s, 2H), 4.56 (s, 2H), 3.88 (s, 3H) ppm. **^{13}C NMR** (101 MHz, $CDCl_3$) δ 149.3, 148.3, 137.1, 133.6, 128.6, 127.9, 127.4, 120.1, 113.2, 111.8, 71.0, 65.2, 56.1 ppm. The data is consistent with literature.(Nawrat et al., 2013)

2p

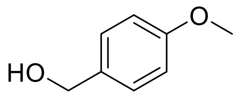

Prepared according to the general procedure. The crude product was purified by silica gel column chromatography (pentane:diethyl ether, 100:5 → 100:20) to obtain the desired product as a white solid (35 mg, 51 %). **^{1}H NMR** (400 MHz, $CDCl_3$) δ 7.28 (d, J = 8.5 Hz, 2H), 6.89 (d, J = 8.6 Hz, 2H), 4.60 (s, 2H), 3.80 (s, 3H) ppm. **^{13}C NMR** (101 MHz, $CDCl_3$) δ 159.4, 133.3, 128.8, 114.1, 65.2, 55.4. The data is consistent with literature.(Shi et al., 2012)

2q

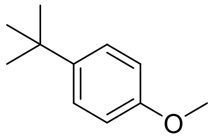

Prepared according to the general procedure. The crude product was purified by silica gel column chromatography (pentane:diethyl ether, 5:1) to obtain the desired product as a clear oil (23 mg, 14 %).**^{1}H NMR** (400 MHz, $CDCl_3$) δ 7.32 (m, 2H), 6.68 (m, 2H), 3.80 (s, 3H), 1.31 (s, 9H). **^{13}C NMR** (101 MHz, $CDCl_3$) δ 157.3, 143.3, 126.2, 113.4, 55.2, 34.1, 31.6. The data is consistent with literature.(Cheung & Buchwald, 2013)

2r

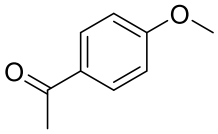

Prepared according to the general procedure. The crude product was purified by silica gel column chromatography (heptane:EtOAc, 100:10) to obtain the desired product as a white solid (33 mg, 29 %). **^{1}H NMR** (400 MHz, $CDCl_3$) δ 7.98 – 7.90 (m, 2H), 6.97 – 6.90 (m, 2H), 3.87 (s, 3H), 2.55 (s, 3H). **^{13}C NMR** (101 MHz, $CDCl_3$) δ 196.9, 163.6, 130.7, 130.5, 113.8, 55.6, 26.5. The data is consistent with the literature.(Zhang et al., 2012)

2s

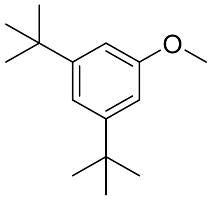

Prepared according to the general procedure. The crude product was purified by silica gel column chromatography (pentane:diethyl ether, 100:0 → 100:10) to obtain the desired product as a clear oil (21 mg, 19 %). **^{1}H NMR** (400 MHz, $CDCl_3$) δ 7.03 (t, *J* = 1.6 Hz, 1H), 6.77 (d, *J* = 1.7 Hz, 2H), 3.82 (s, 3H), 1.33 (s, 18H). **^{13}C NMR** (101 MHz, $CDCl_3$) δ 159.2, 152.4, 115.1, 108.4, 55.3, 35.1, 31.6. The data is consistent with literature.(Sun et al., 2020)

2t

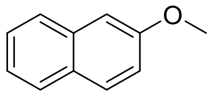

Prepared according to the general procedure. The crude product was purified by silica gel column chromatography (pentane:diethyl ether, 4:1) to obtain the desired product as a beige powder (155 mg, 98 %). **^{1}H NMR** (400 MHz, $CDCl_3$) δ 7.76 (dd, *J* = 11.6, 8.4 Hz, 3H), 7.44 (t, *J* = 7.5 Hz, 1H), 7.34 (t, *J* = 7.5 Hz, 1H), 7.15 (d, *J* = 7.4 Hz, 2H), 3.93 (s, 3H). **^{13}C NMR** (100 MHz, $CDCl_3$): δ 157.6, 134.5, 129.4, 129.0, 127.7, 126.8, 126.4, 123.6, 118.7, 105.8, 55.3. The data is consistent with literature.(Shen et al., 2025)

2u

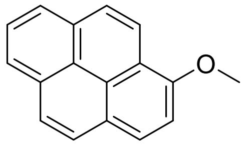

Prepared according to the general procedure. The crude product was purified by silica gel column chromatography (pentane:diethyl ether, 100:5) to obtain the desired product as a green solid (155 mg, 89 %). **^{1}H NMR** (400 MHz, $CDCl_3$) δ 8.47 (d, *J* = 9.2 Hz, 1H), 8.16 – 8.02 (m, 4H), 8.00 – 7.94 (m, 2H), 7.89 (d, *J* = 8.9 Hz, 1H), 7.55 (d, *J* = 8.4 Hz, 1H), 4.18 (s, 3H). **^{13}C NMR** (101 MHz, $CDCl_3$) δ 153.8, 131.9, 131.8, 127.4, 126.5, 126.2, 126.0, 125.6, 125.4, 125.1, 125.1, 124.4, 124.3, 121.3, 120.4, 108.2, 56.3. The data is consistent with the literature.(Tang et al., 2018)

2v

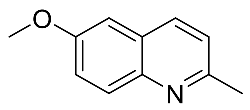

Prepared according to the general procedure. The crude product was purified by silica gel column chromatography (pentane:diethyl ether, 10:1 → 2:1) to obtain the desired product as a colorless solid (130 mg, 81 %). R_f = 0,57 (pentane:diethyl ether, 1:1). **^{1}H NMR** (400 MHz, $CDCl_3$) δ 7.92–7.86 (m, 2H), 7.31 (dd, *J* = 9.1, 2.8 Hz, 1H), 7.18 (d, *J* = 8.4 Hz, 1H), 6.99 (d, *J* = 2.8 Hz, 1H), 3.86 (s, 3H), 2.67 (s, 3H). **^{13}C NMR** (100 MHz, $CDCl_3$) δ 157.1, 156.3, 143.9, 135.0, 130.0, 127.3, 121.8, 121.2, 106.2, 55.4, 25.0. **IR (cm^{-1})** ν 3004, 2952, 2841, 1627, 1604, 1501, 1235, 1164, 1022. **HR-MS** (+APCI) *m/z* calculated for $C_{11}H_{12}NO^+$ $[M+H]^+$: 174.0913; found: 174.0913. The data is consistent with literature.(He et al., 2017)

2w

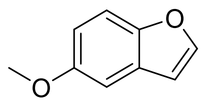

Prepared according to the general procedure. The crude product was purified by silica gel column chromatography (pentane:diethyl ether, 100:0 → 6:1) to obtain the desired product as a colorless solid (30 mg, 20 %). R_f = 0,19 (pentane). **^{1}H NMR** (400 MHz, $CDCl_3$) δ 7.60 (d, *J* = 2.2 Hz, 1H), 7.40 (d, *J* = 8.4 Hz, 1H), 7.06 (d, *J* = 2.6 Hz, 1H), 6.91 (dd, *J* = 8.9, 2.6 Hz, 1H), 6.71 (dd, *J* = 2.2, 1.1 Hz, 1H), 3.85 (s, 3H). **^{13}C NMR** (100 MHz, $CDCl_3$) δ 156.0, 150.0, 145.7, 128.0, 113.1, 111.8, 106.7, 103.7, 55.9. The data is

2x

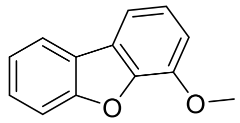

Prepared according to the general procedure. The crude product was purified by silica gel column chromatography (pentane:diethyl ether, 1:6) to obtain the desired product as a colorless solid (75 mg, 38 %). R_f = 0,57 (pentane:diethyl ether, 1:6). **^{1}H NMR** (400 MHz, $CDCl_3$) δ 7.94 (d, *J* = 7.7 Hz, 1H, H-8), 7.63 (d, *J* = 8.3 Hz, 1H, H-11), 7.56 (d, *J* = 7.8 Hz, 1H, H-5), 7.46 (t, *J* = 8.0 Hz, 1H, H-10), 7.35 (t, *J* = 7.5 Hz, 1H, H-9), 7.28 (t, *J* = 7.5 Hz, 1H, H-4), 7.00 (d, *J* = 8.0 Hz, 1H, H-3), 4.08 (s, 3H, H-1). **^{13}C NMR** (100 MHz, $CDCl_3$) δ 156.2, 145.8, 145.2, 127.2, 125.5, 124.4, 123.4, 122.8, 120.8, 112.8, 112.0, 109.4, 56.2. **IR (cm^{-1})** 3058, 2843, 1905, 1640, 1503, 1454, 1428, 1335, 1313, 1272, 1182. **HR-MS** (+APCI) *m/z* calculated for $C_{13}H_{10}O_2^+$ $[M+H]^+$: 198.0681; found: 198.0681. The data is consistent with literature.(Asahara et al., 2019)

5. Spectral information

Data for 2a

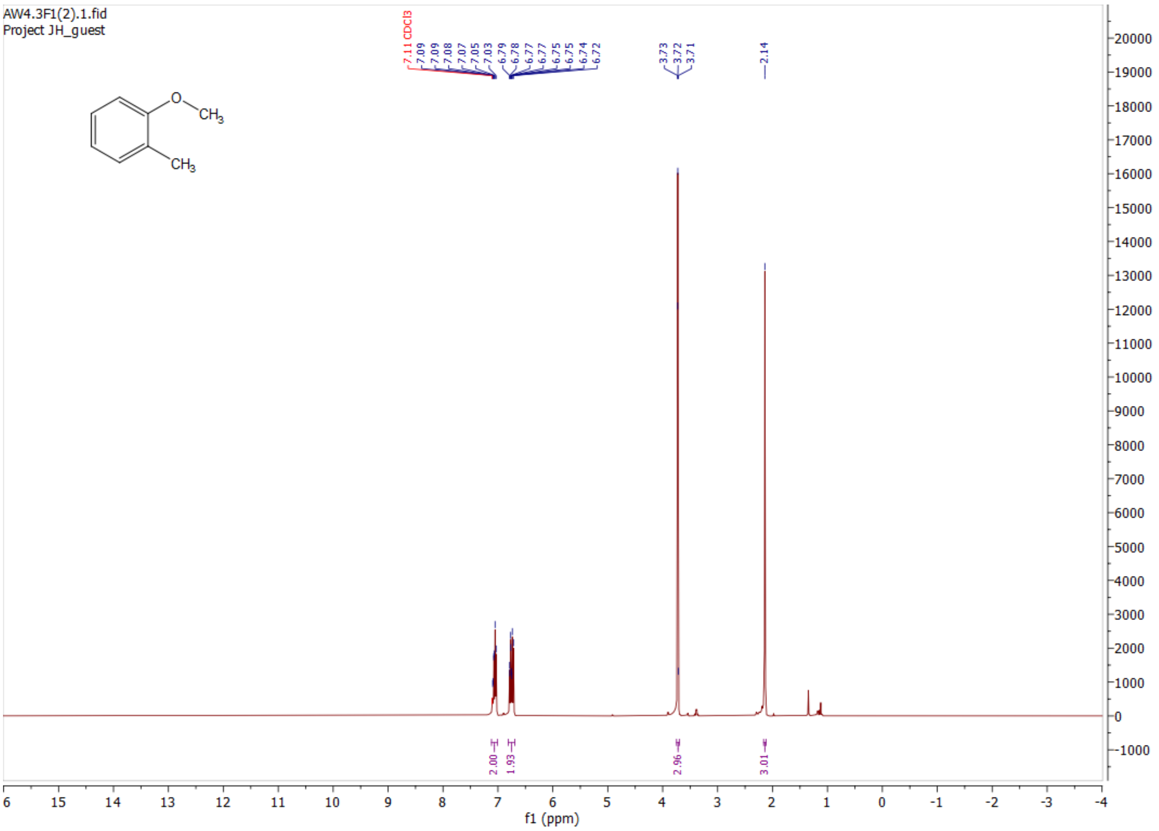

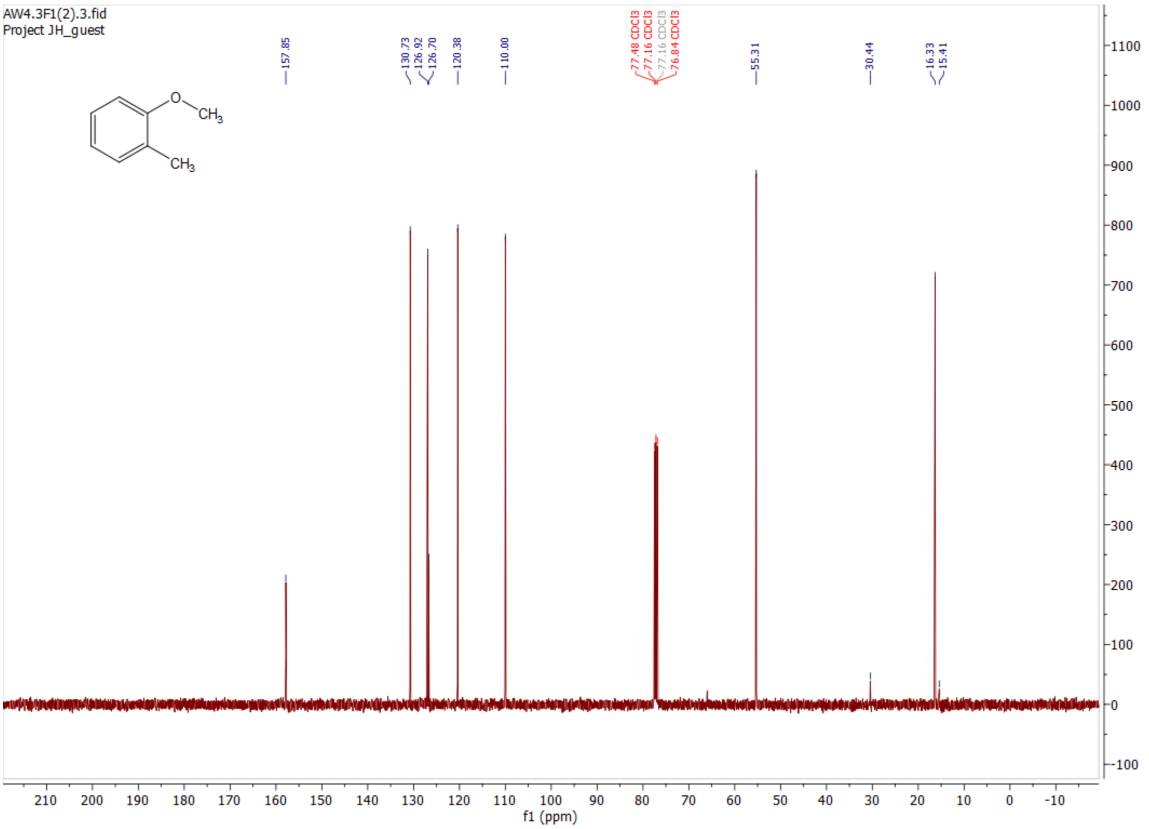

Data for 2b

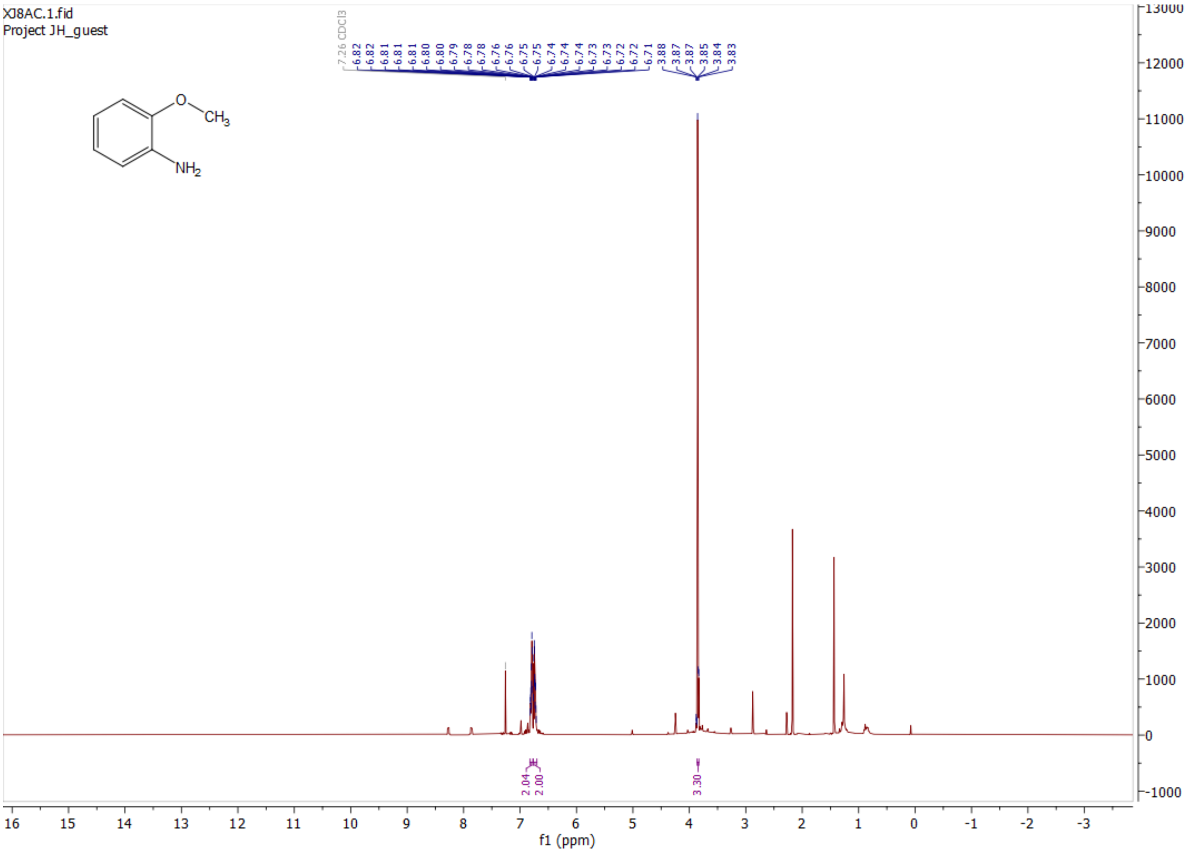
XJ8AC.1.fid
Project JH_guest
O
CH3
NH2
f1 (ppm)

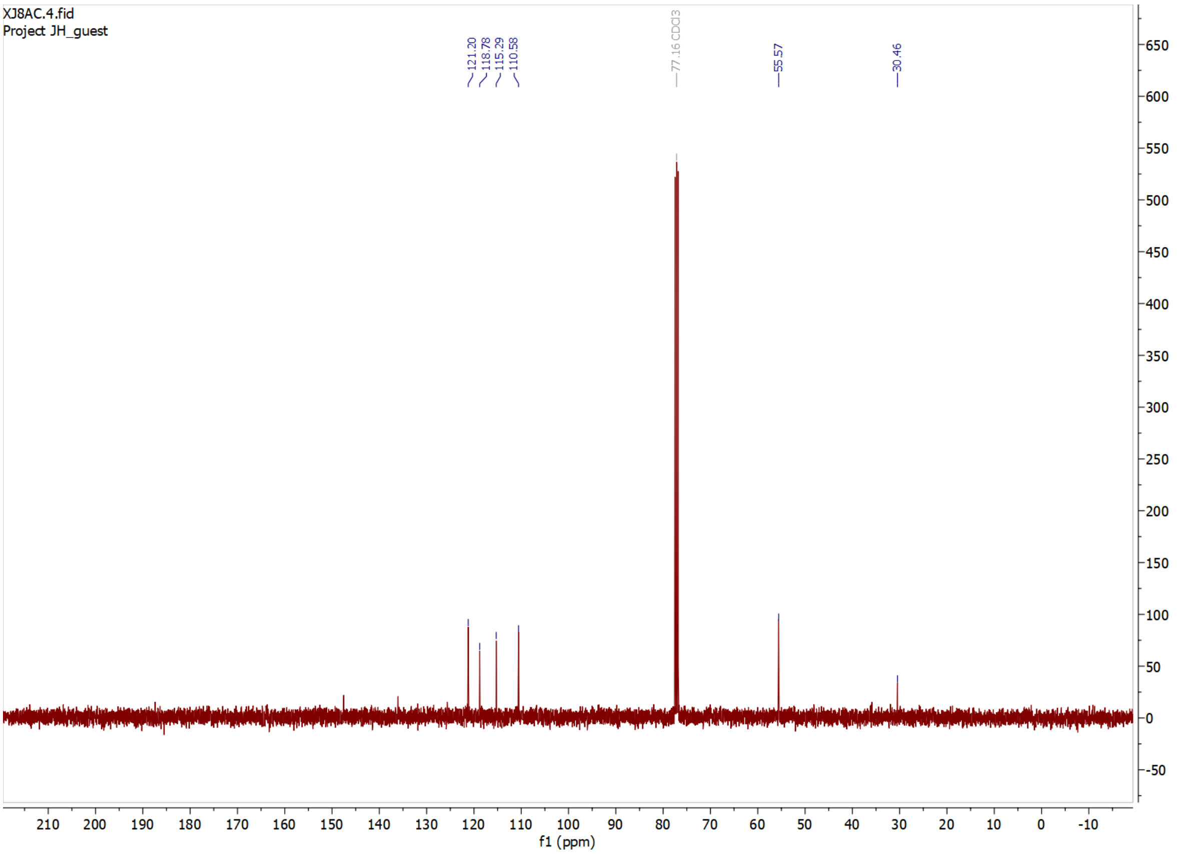
XJ8AC.4.fid
Project JH_guest
121.20
118.78
115.29
110.58
77.16 CDCl3
55.57
30.46
f1 (ppm)

Data for 2c

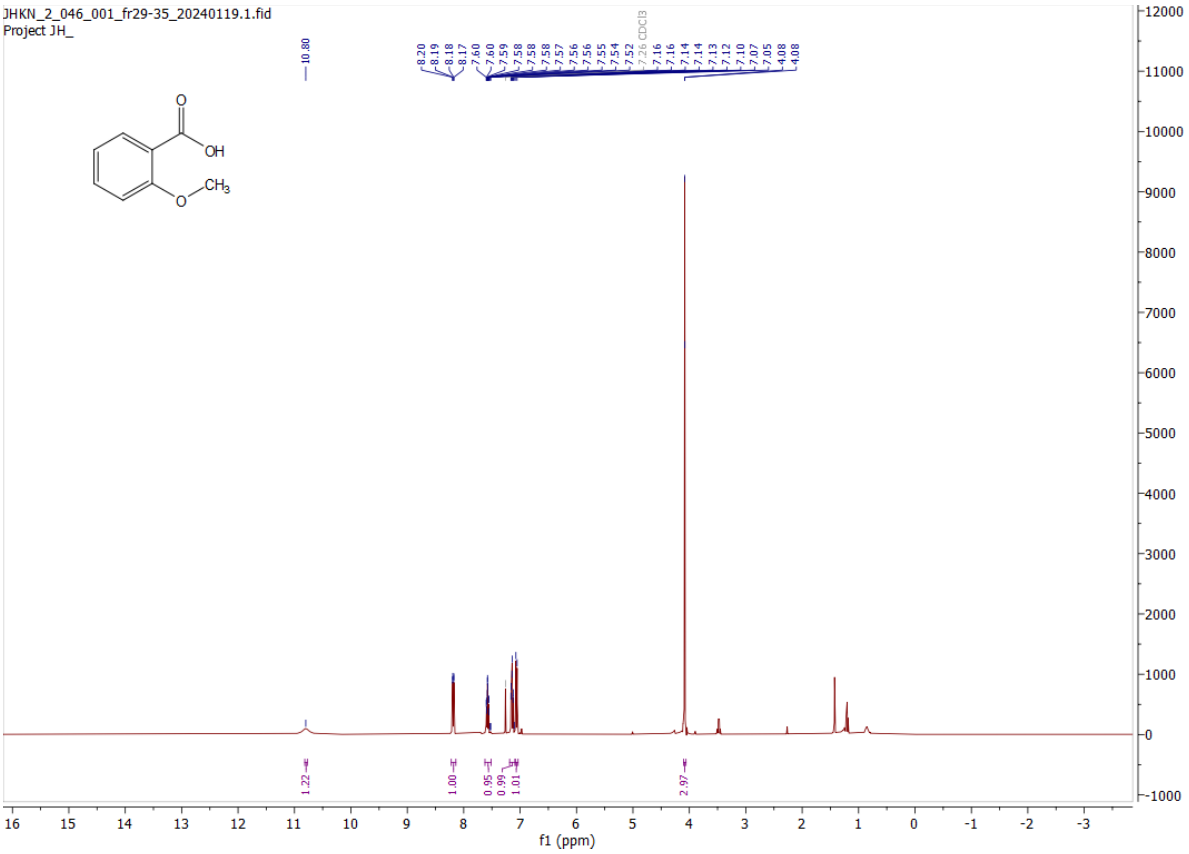
JHKN_2_046_001_fr29-35_20240119.1.fid
Project JH_
10.80
8.20 8.19 8.18 8.17 7.60 7.59 7.58 7.58 7.58 7.57 7.56 7.56 7.55 7.54 7.52 7.26 CDCl3 7.16 7.16 7.14 7.14 7.13 7.12 7.10 7.07 7.05 4.08 4.08
O
OH
O
CH3
1.22
1.00
0.95
0.99
1.01
2.97
f1 (ppm)

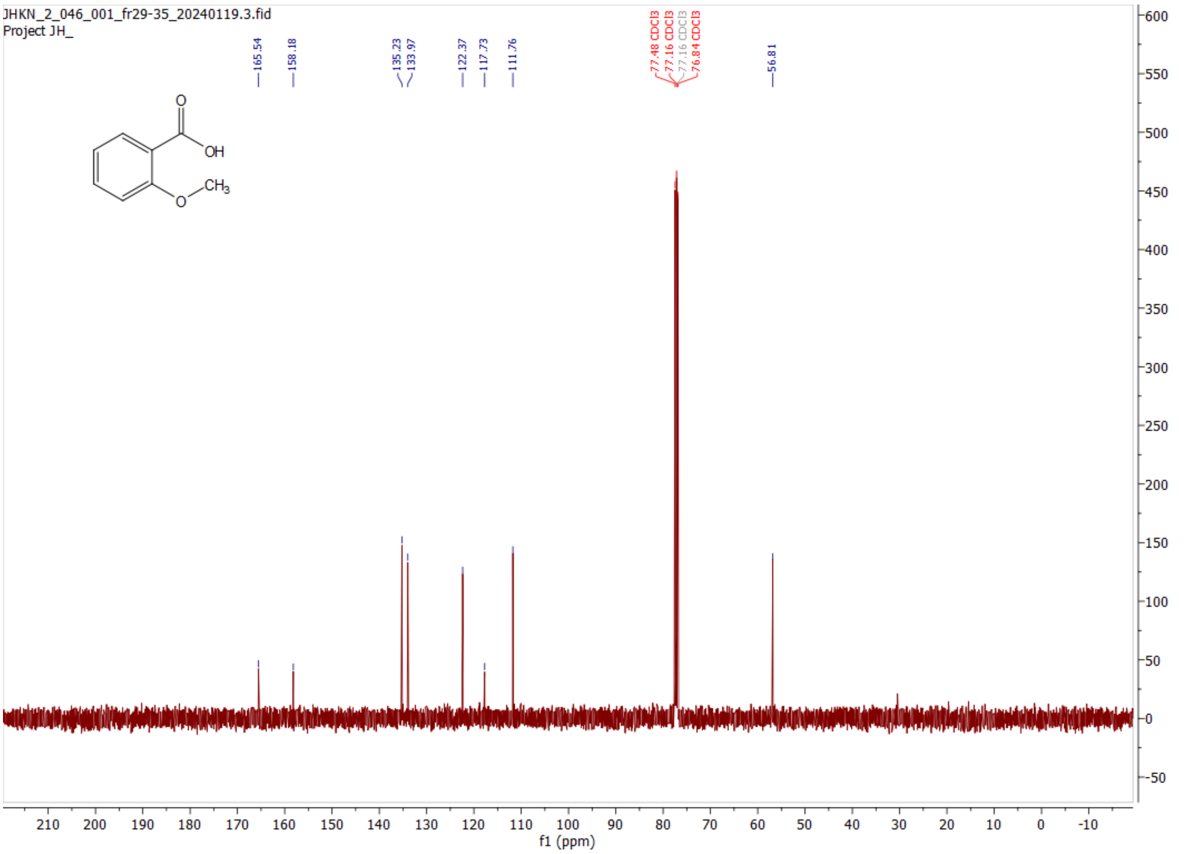
JHKN_2_046_001_fr29-35_20240119.3.fid
Project JH_
165.54
158.18
135.23
133.97
122.37
117.73
111.76
77.48 CDCl3
77.16 CDCl3
77.16 CDCl3
76.84 CDCl3
56.81
O
OH
O
CH3
f1 (ppm)

Data for 2d

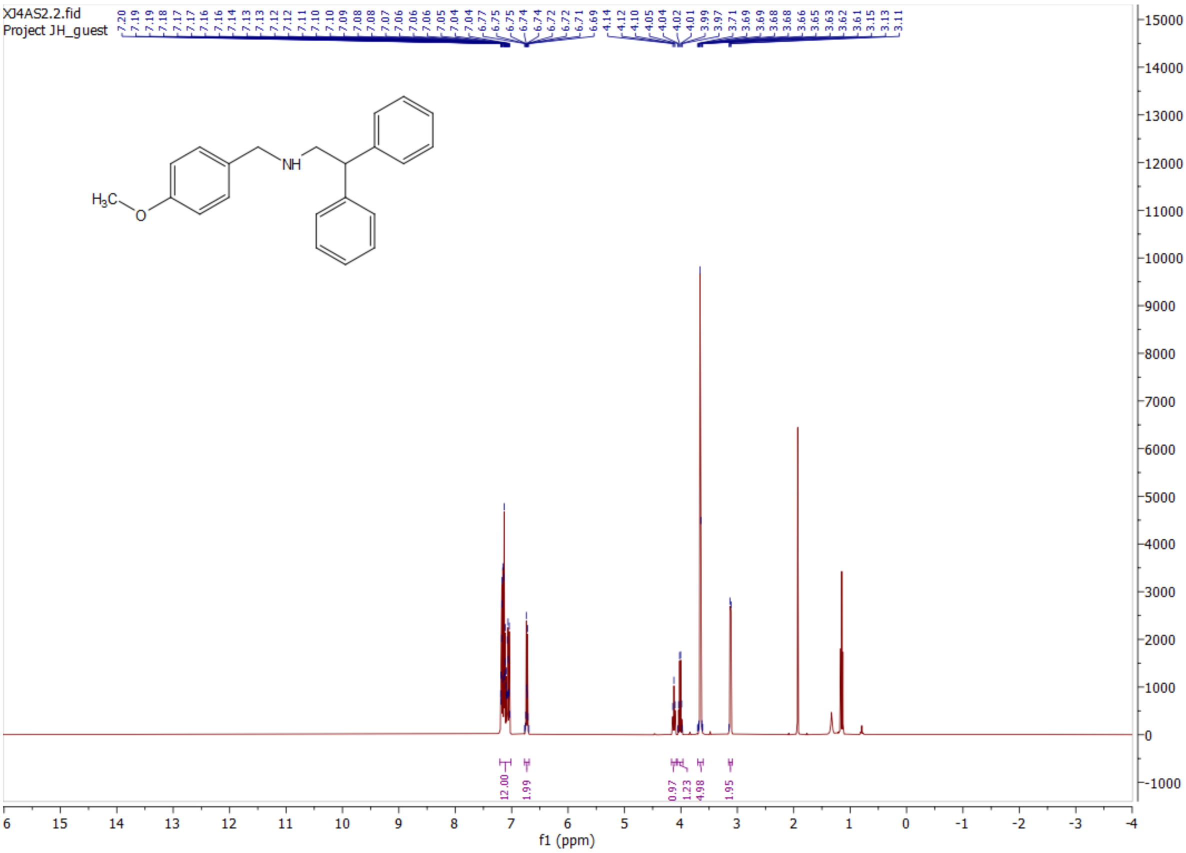
XJ4AS2.2.fid
Project JH_guest
NH
H3C
O
12.00
1.99
0.97
1.23
4.98
1.95
f1 (ppm)

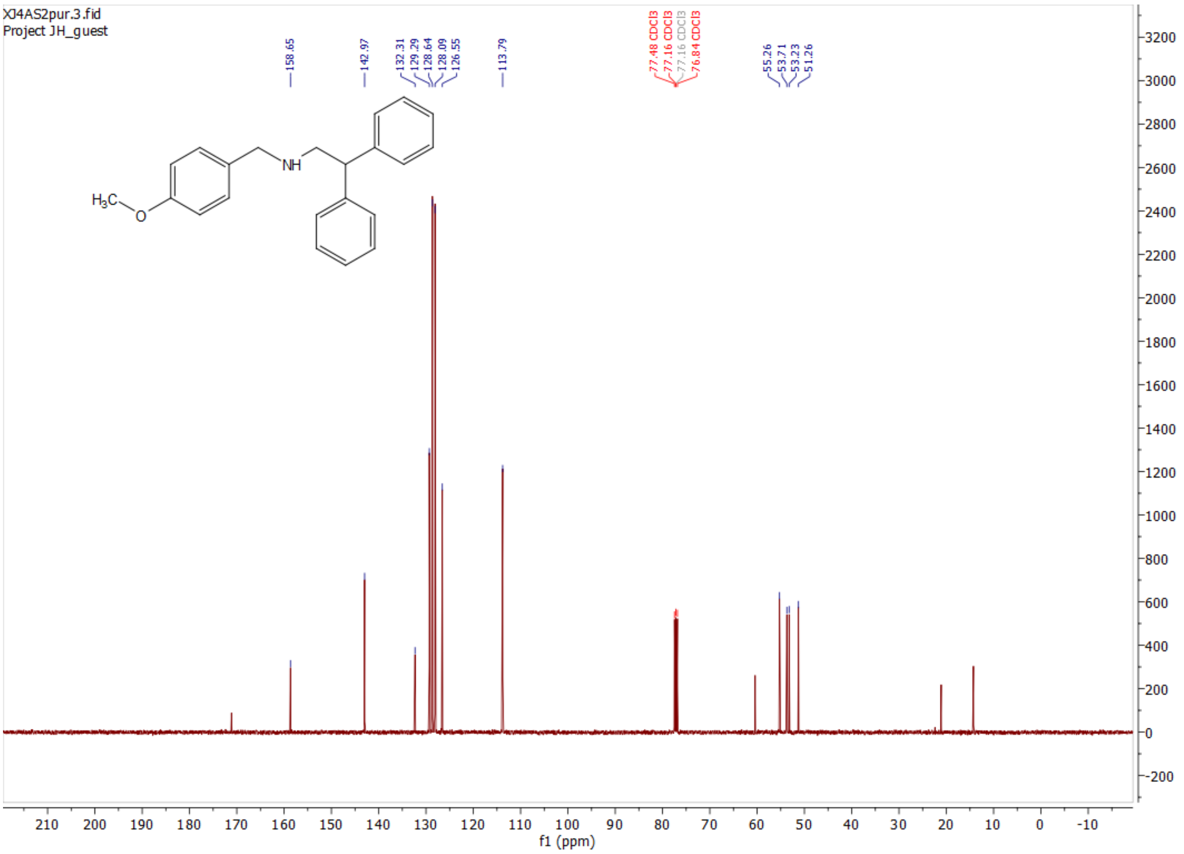
XJ4AS2pur.3.fid
Project JH_guest
158.65
142.97
132.31
129.29
128.64
128.09
126.55
113.79
77.48 CDCl3
77.16 CDCl3
77.16 CDCl3
76.84 CDCl3
55.26
53.71
53.23
51.26
NH
H3C
O
f1 (ppm)

Data for 2e

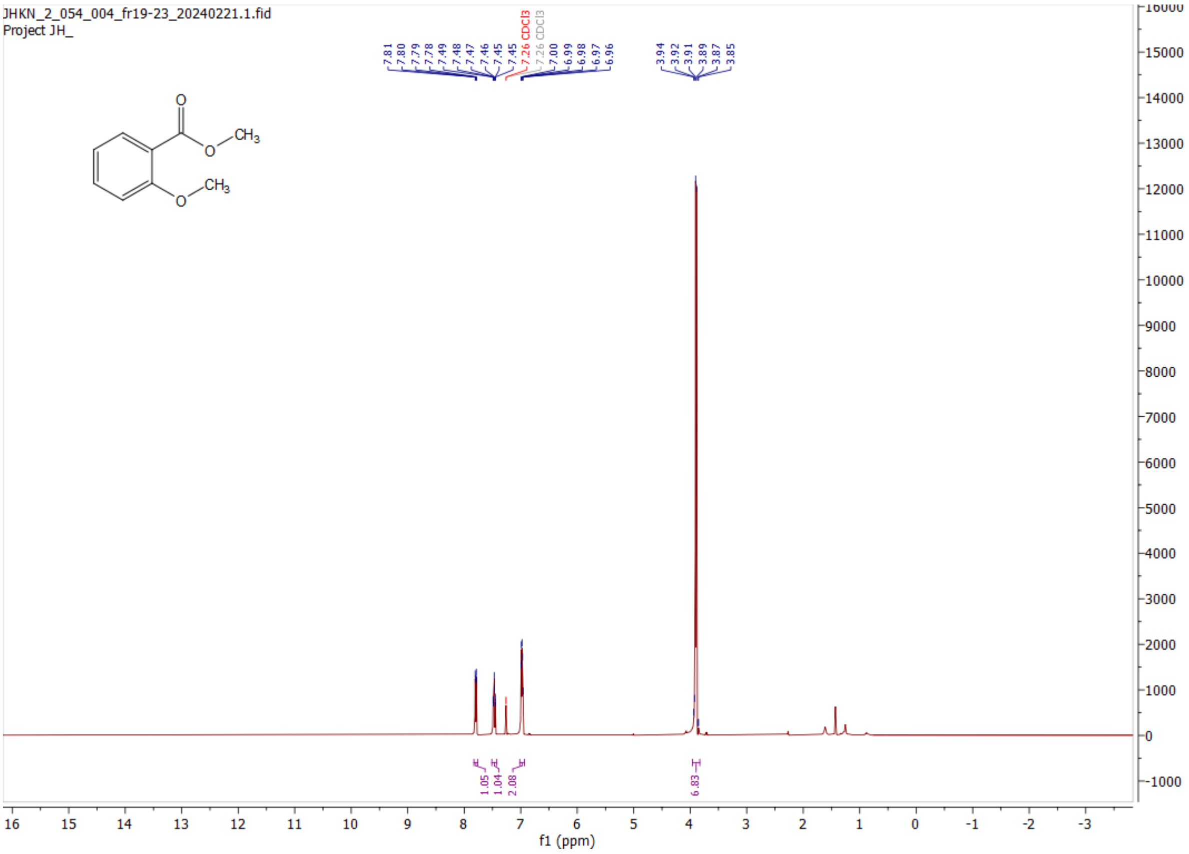

JHKN_2_054_004_fr19-23_20240221.1.fid
Project JH_
7.81
7.80
7.79
7.78
7.49
7.48
7.47
7.46
7.45
7.45
7.26 CDCl3
7.26 CDCl3
7.00
6.99
6.98
6.97
6.96
3.94
3.92
3.91
3.89
3.87
3.85
1.05
1.04
2.08
6.83
f1 (ppm)

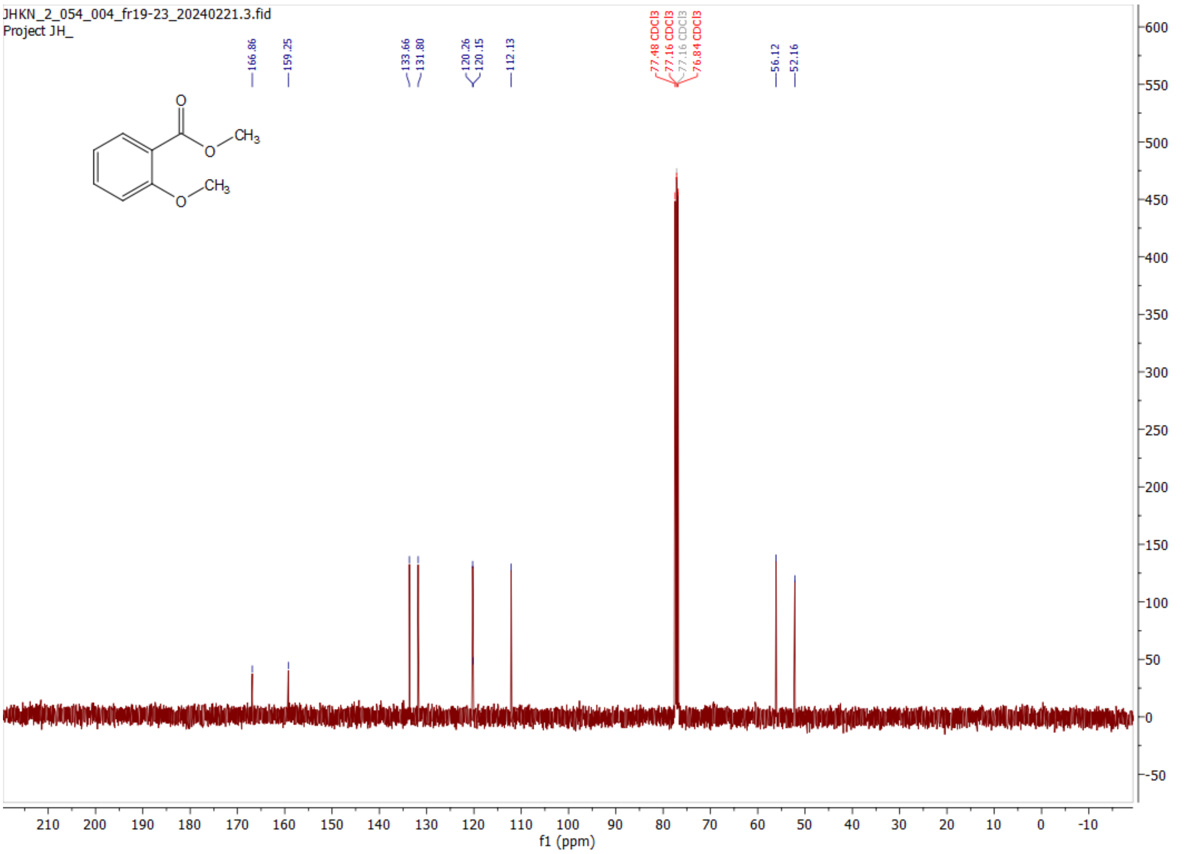

JHKN_2_054_004_fr19-23_20240221.3.fid
Project JH_
166.86
159.25
133.66
131.80
120.26
120.15
112.13
77.48 CDCl3
77.16 CDCl3
77.16 CDCl3
76.84 CDCl3
56.12
52.16
f1 (ppm)

Data for 2f

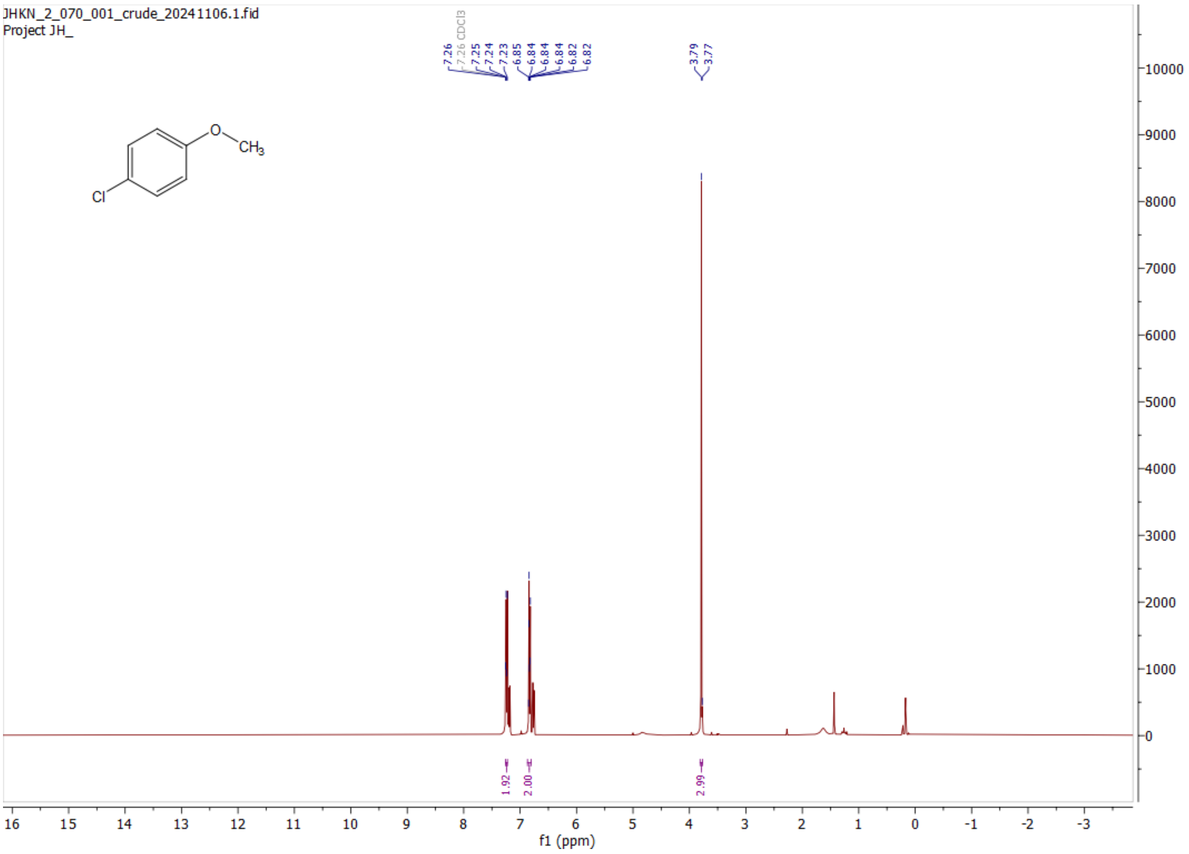
JHKN_2_070_001_crude_20241106.1.fid
Project JH_
7.26
7.26 CDCl3
7.25
7.24
7.23
6.85
6.84
6.84
6.84
6.82
6.82
3.79
3.77
Cl
O
CH3
1.92
2.00
2.99
f1 (ppm)

Data for 2g

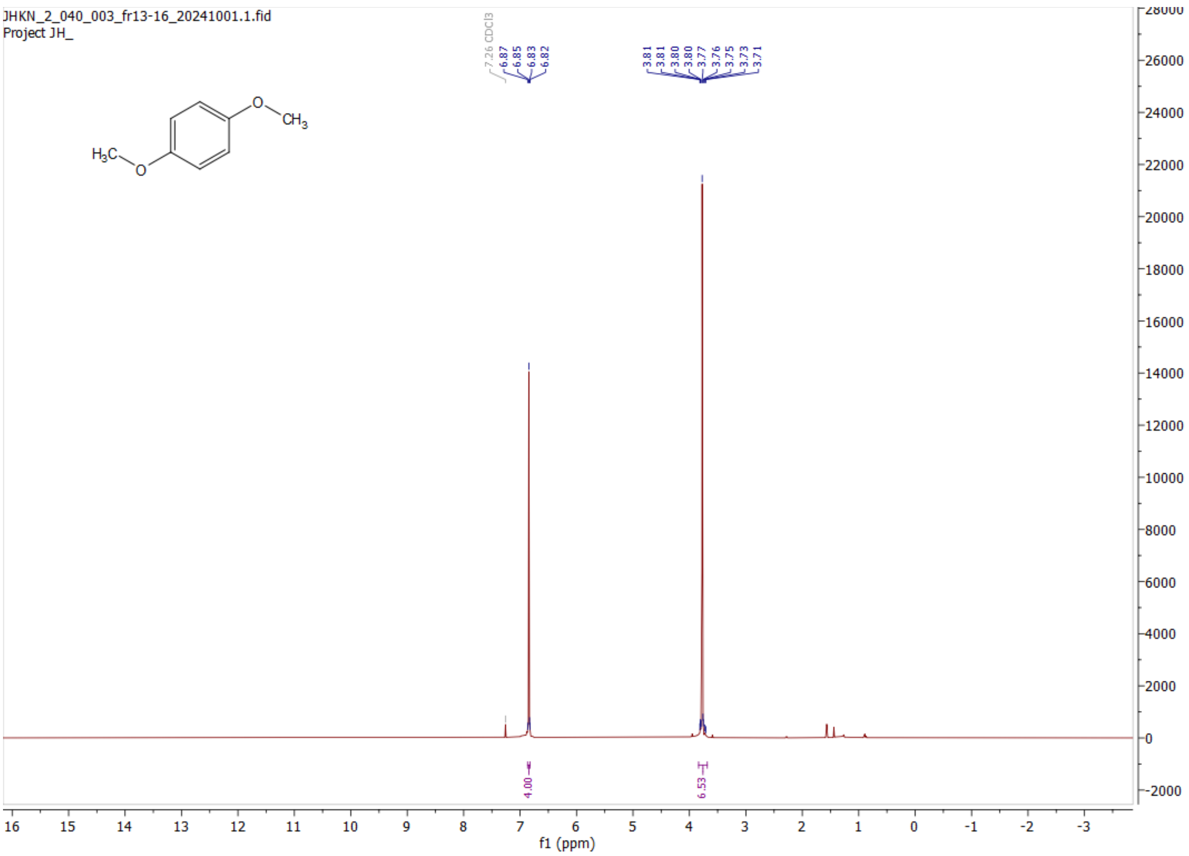

JHKN_2_040_003_fr13-16_20241001.1.fid
Project JH_
7.26 CDCl3
6.87
6.85
6.83
6.82
3.81
3.81
3.80
3.80
3.77
3.76
3.75
3.73
3.71
4.00
6.53
f1 (ppm)

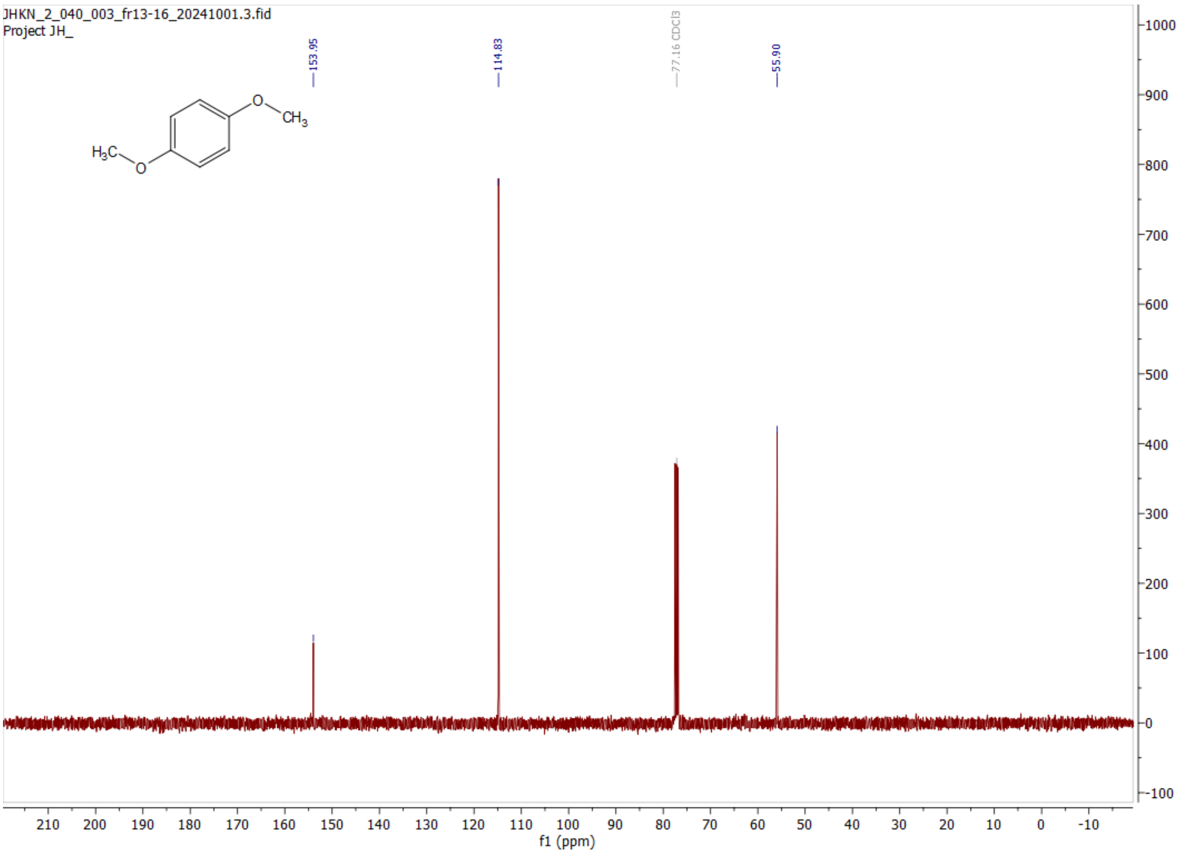

JHKN_2_040_003_fr13-16_20241001.3.fid
Project JH_
153.95
114.83
77.16 CDCl3
55.90
f1 (ppm)

Data for 2h

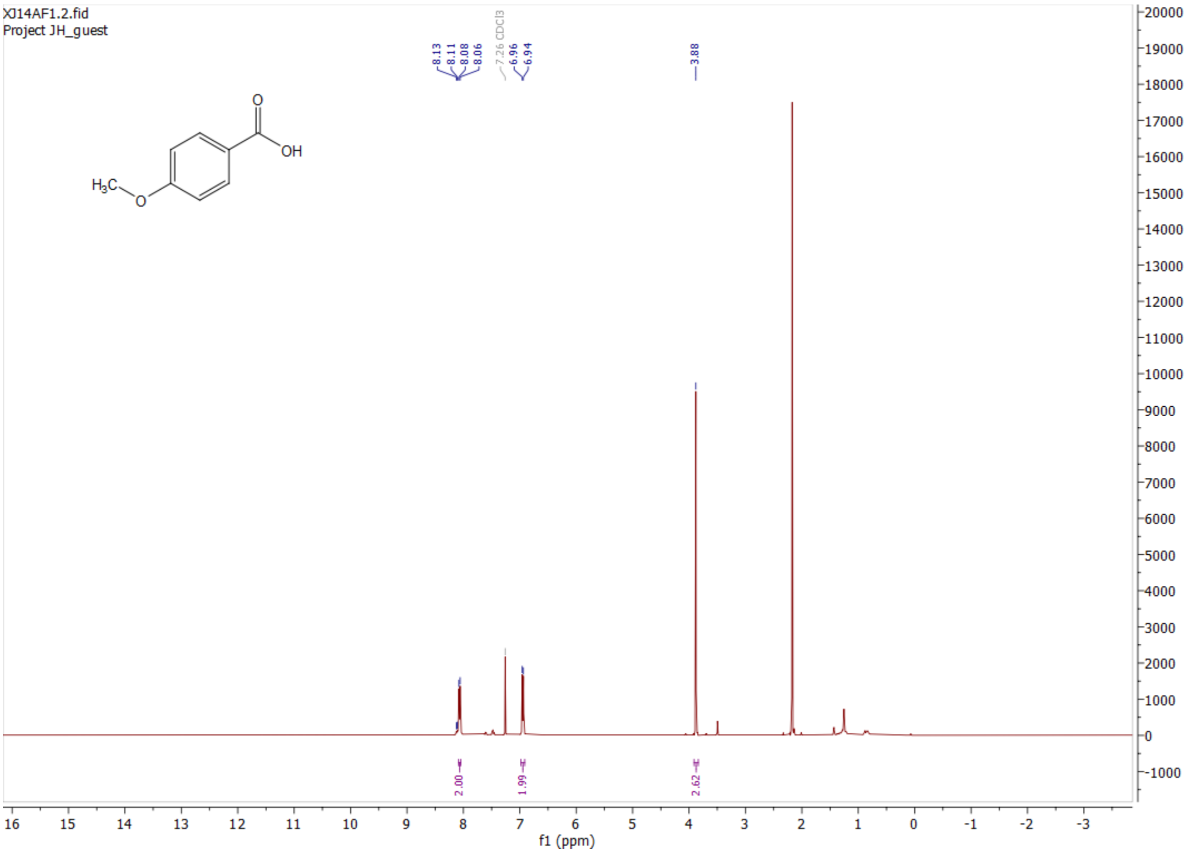
XJ14AF1.2.fid
Project JH_guest
8.13
8.11
8.08
8.06
7.26 CDCl3
6.96
6.94
3.88
OH
O
H3C
O
2.00
1.99
2.62
f1 (ppm)

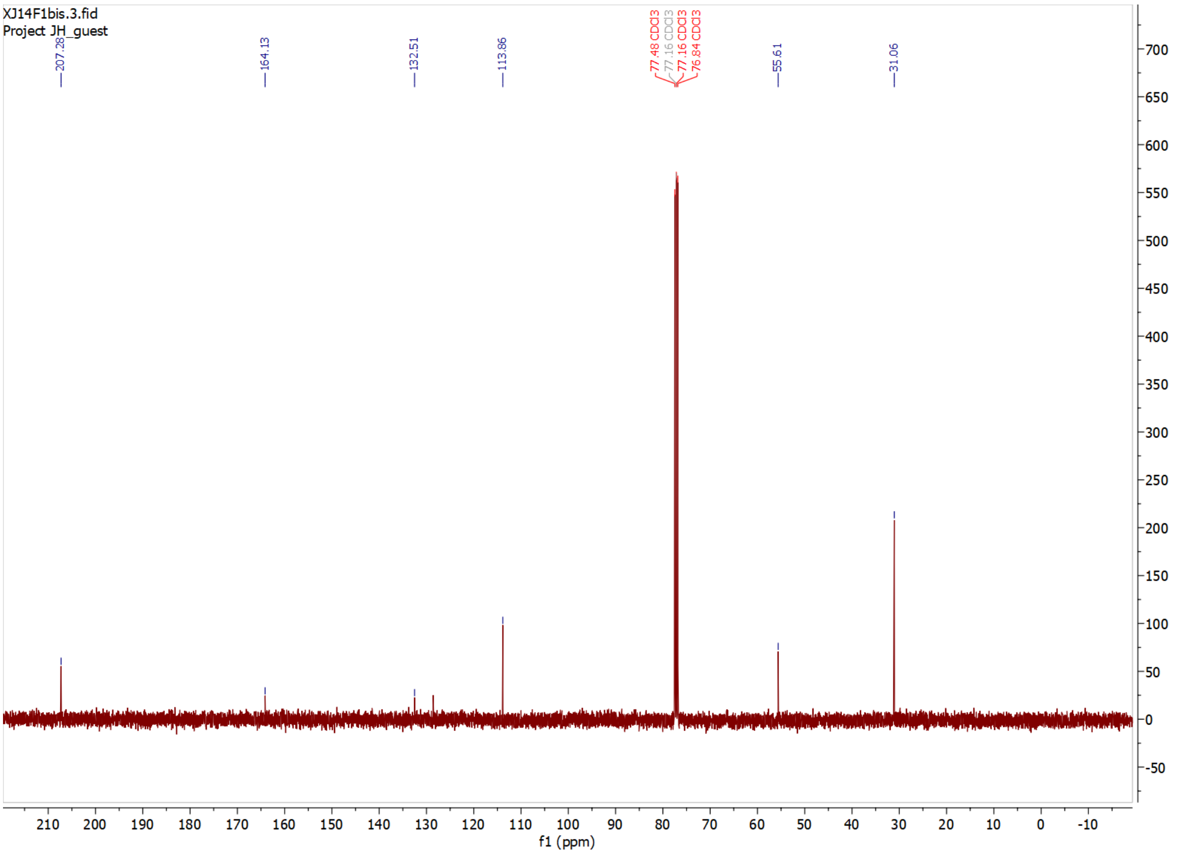
XJ14F1bis.3.fid
Project JH_guest
207.28
164.13
132.51
113.86
77.48 CDCl3
77.16 CDCl3
77.16 CDCl3
76.84 CDCl3
55.61
31.06
f1 (ppm)

Data for 2i

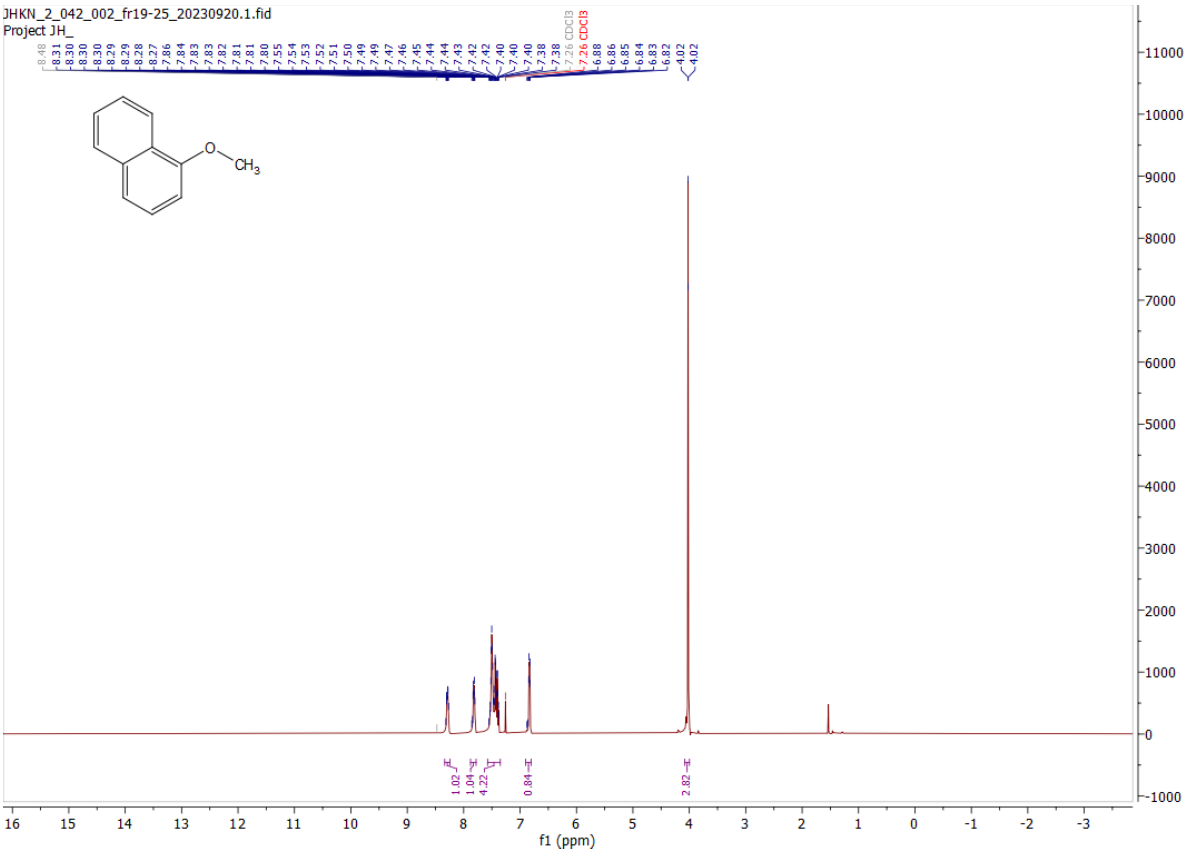

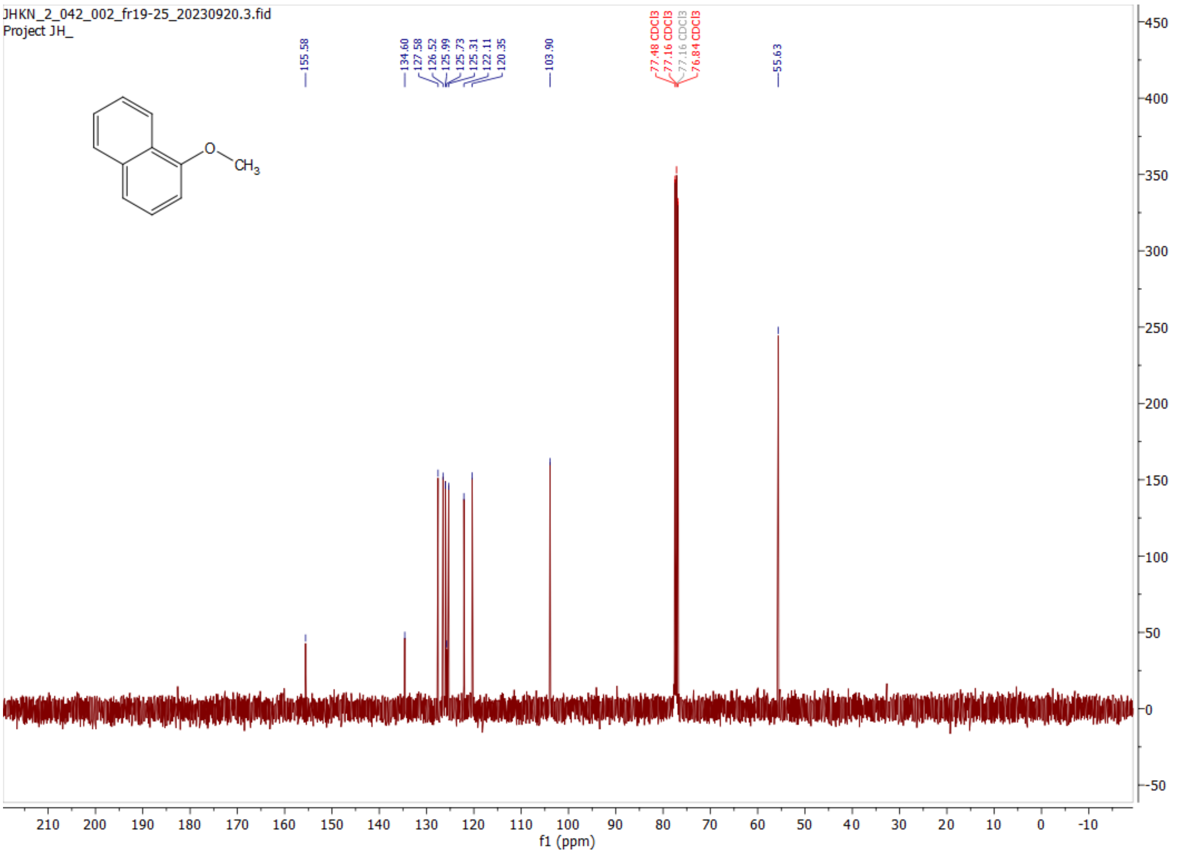

Data for 2j

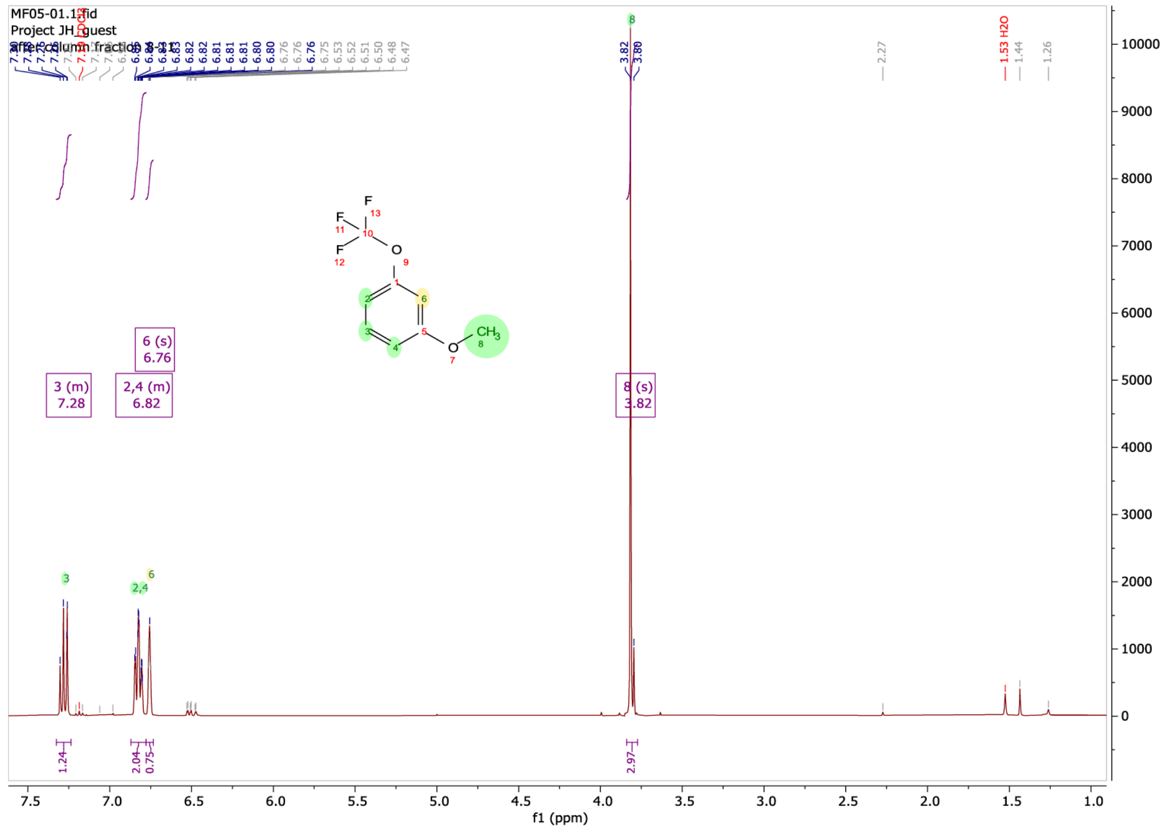

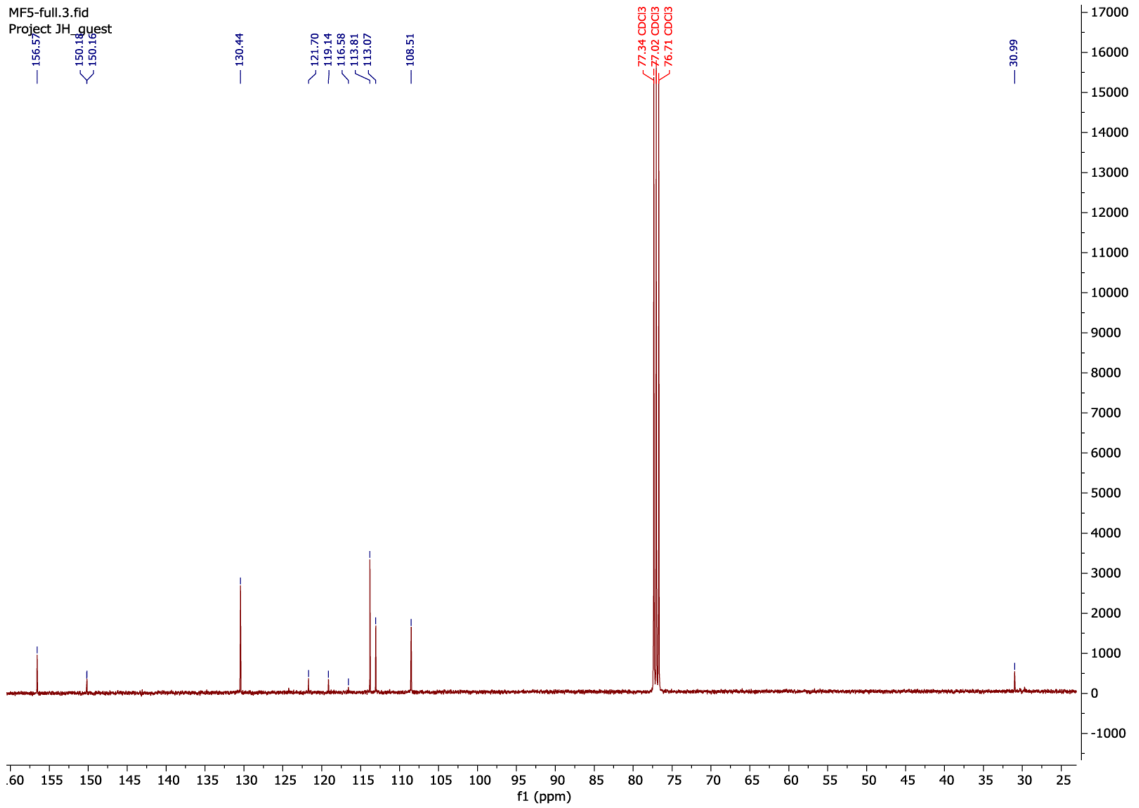

Data for 2k

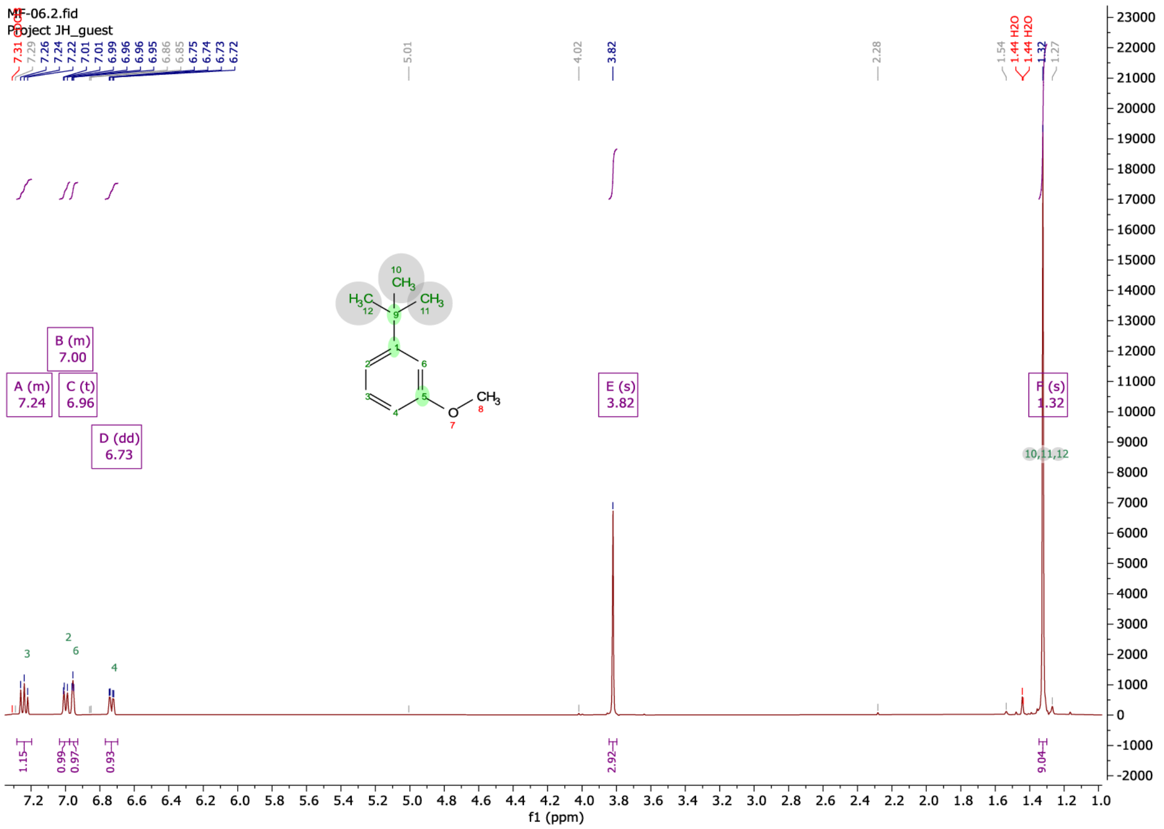

MF-06.2.fid
Project JH_guest
A (m) 7.24
B (m) 7.00
C (t) 6.96
D (dd) 6.73
E (s) 3.82
F (s) 1.32
f1 (ppm)

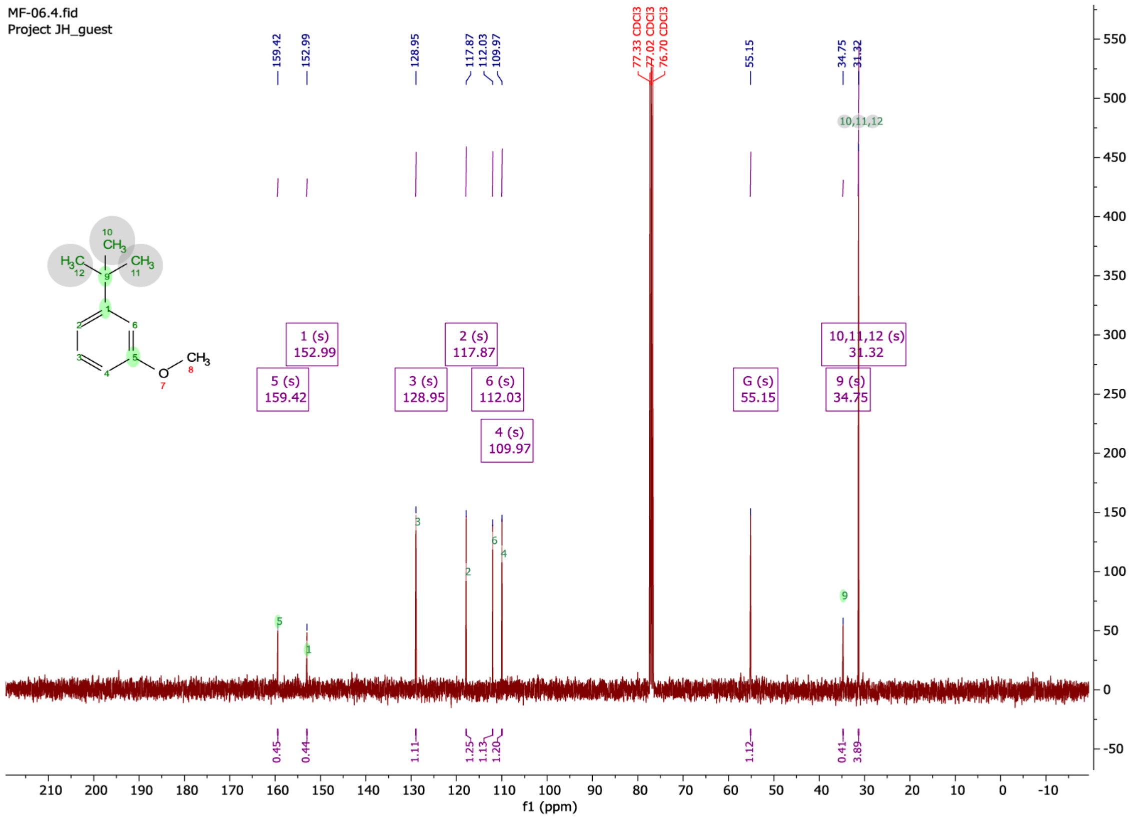

MF-06.4.fid
Project JH_guest
1 (s) 152.99
2 (s) 117.87
3 (s) 128.95
4 (s) 109.97
5 (s) 159.42
6 (s) 112.03
G (s) 55.15
9 (s) 34.75
10,11,12 (s) 31.32
f1 (ppm)

Data for 2l

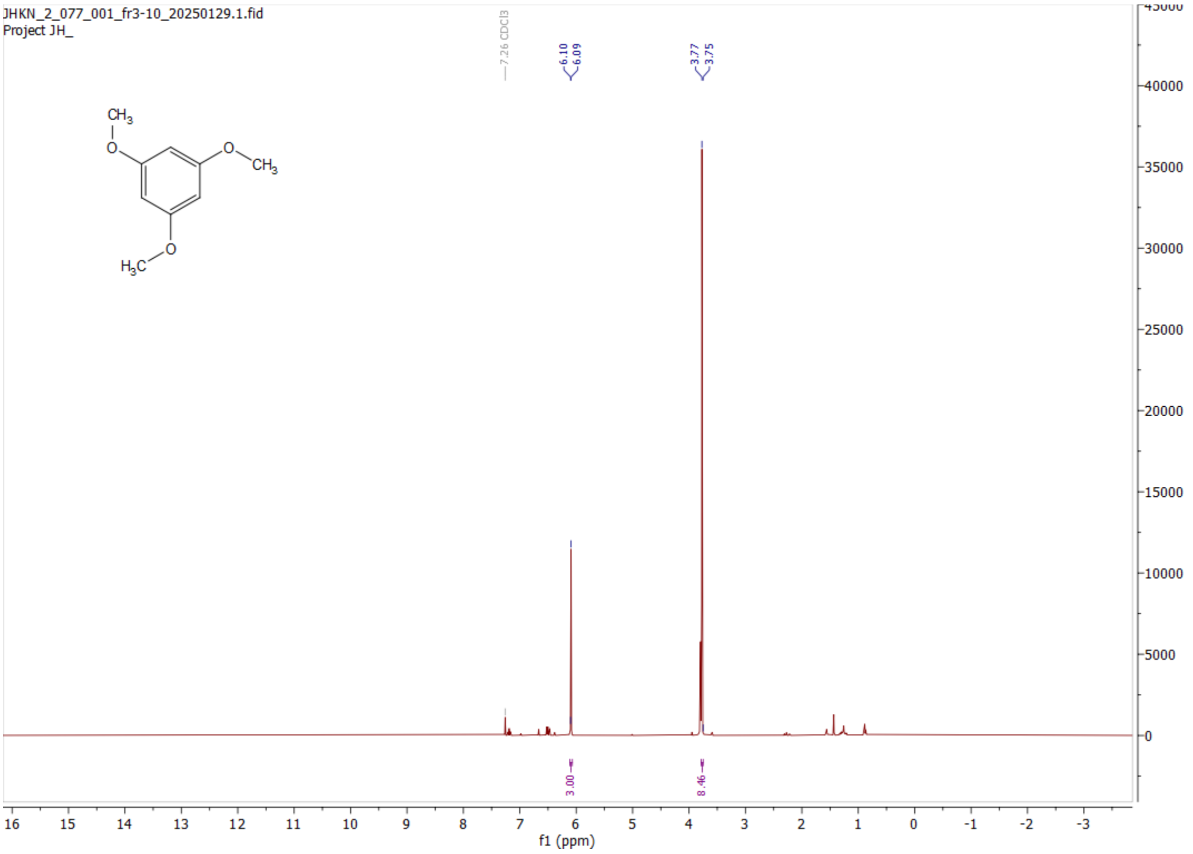

JHKN_2_077_001_fr3-10_20250129.1.fid
Project JH_
7.26 CDCl3
6.10
6.09
3.77
3.75
3.00
8.46
f1 (ppm)

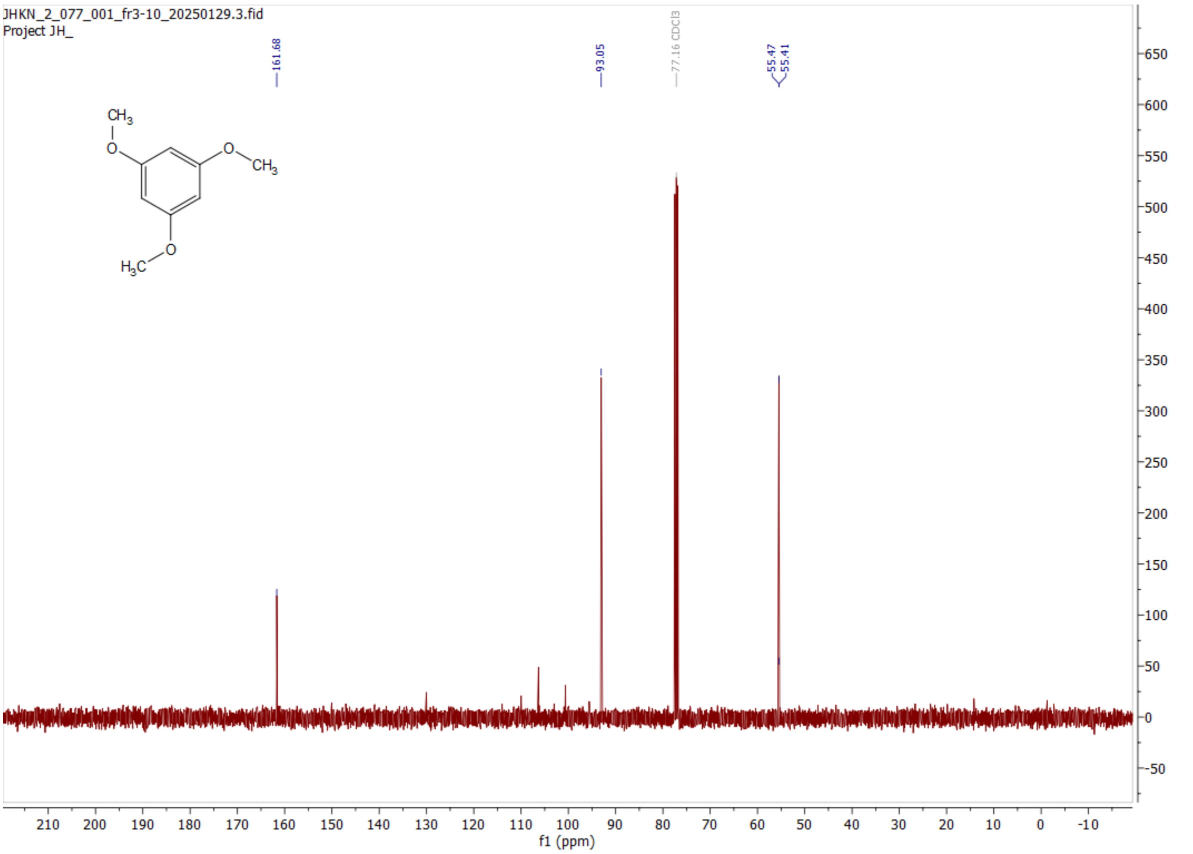

JHKN_2_077_001_fr3-10_20250129.3.fid
Project JH_
161.68
93.05
77.16 CDCl3
55.47
55.41
f1 (ppm)

Data for 2m

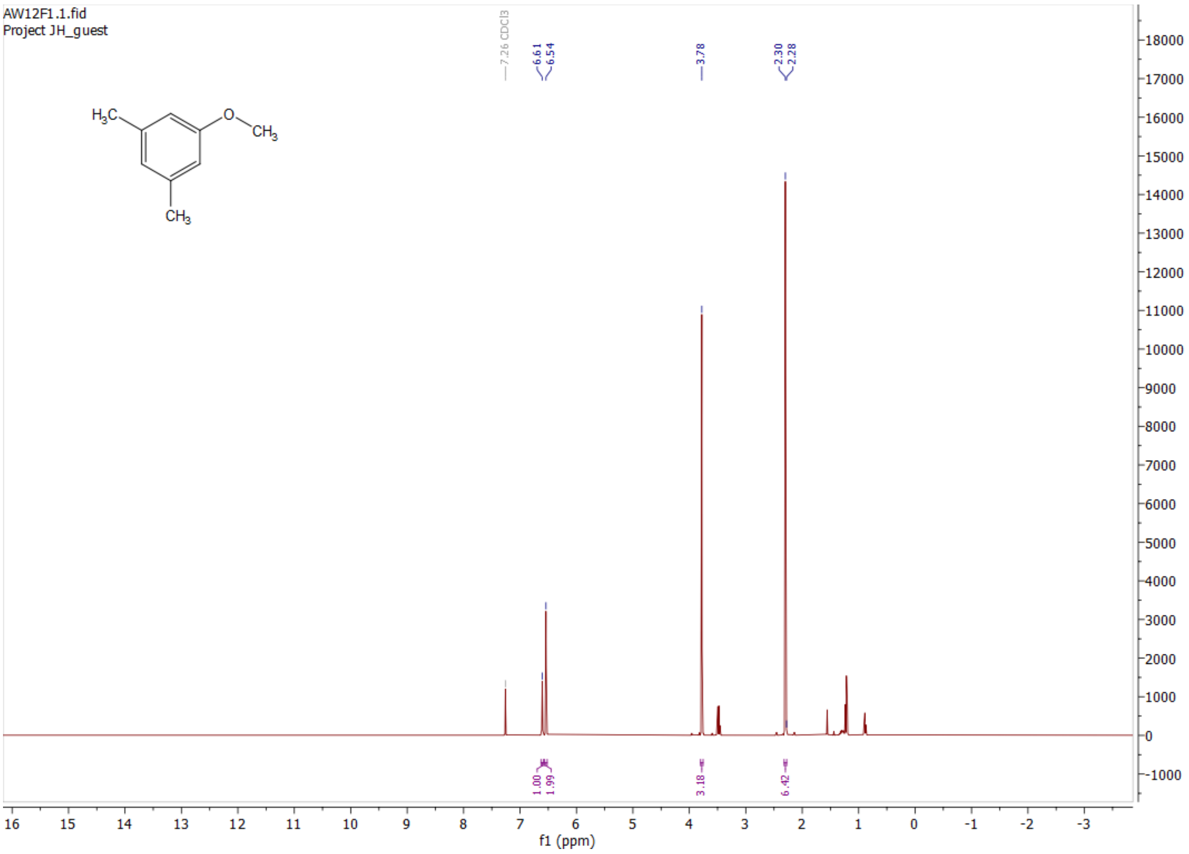
AW12F1.1.fid
Project JH_guest
7.26 CDCl3
6.61
6.54
3.78
2.30
2.28
1.00
1.99
3.18
6.42
f1 (ppm)

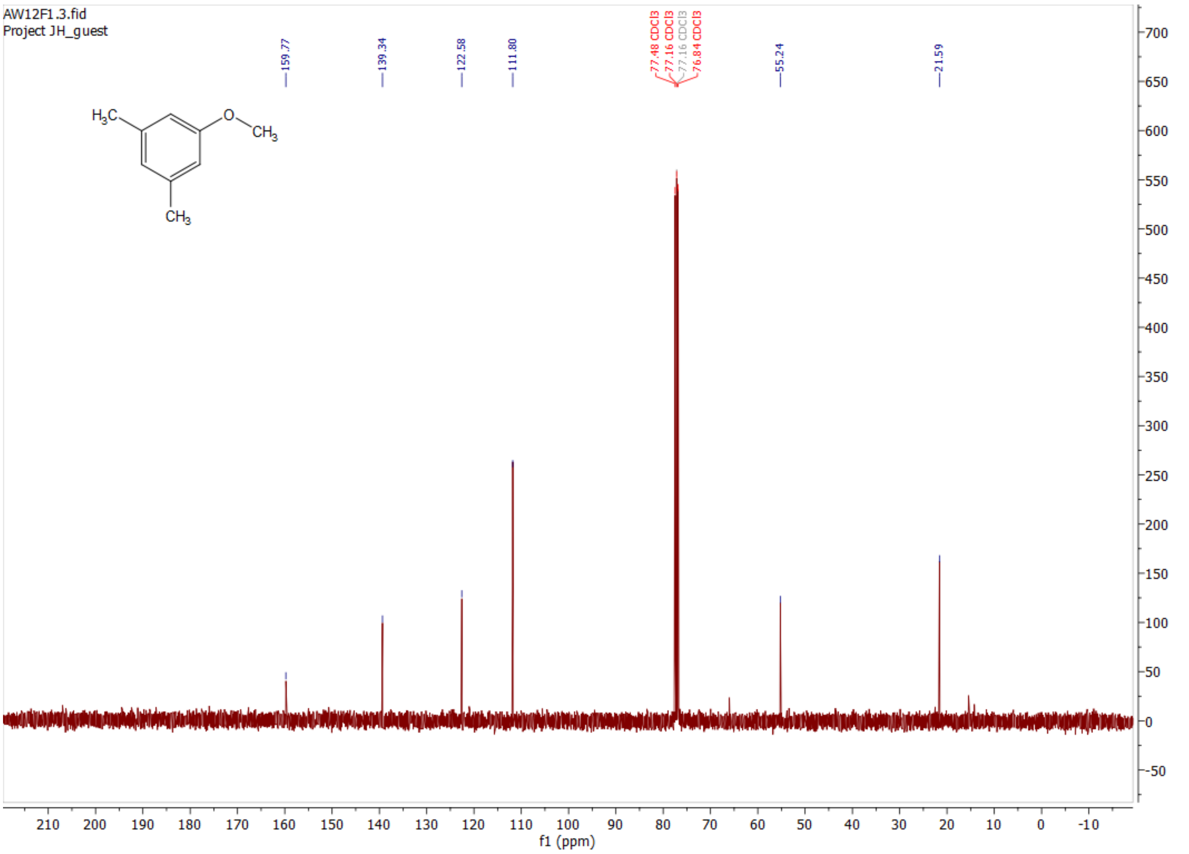
AW12F1.3.fid
Project JH_guest
159.77
139.34
122.58
111.80
77.48 CDCl3
77.16 CDCl3
77.16 CDCl3
76.84 CDCl3
55.24
21.59
f1 (ppm)

Data for 2n

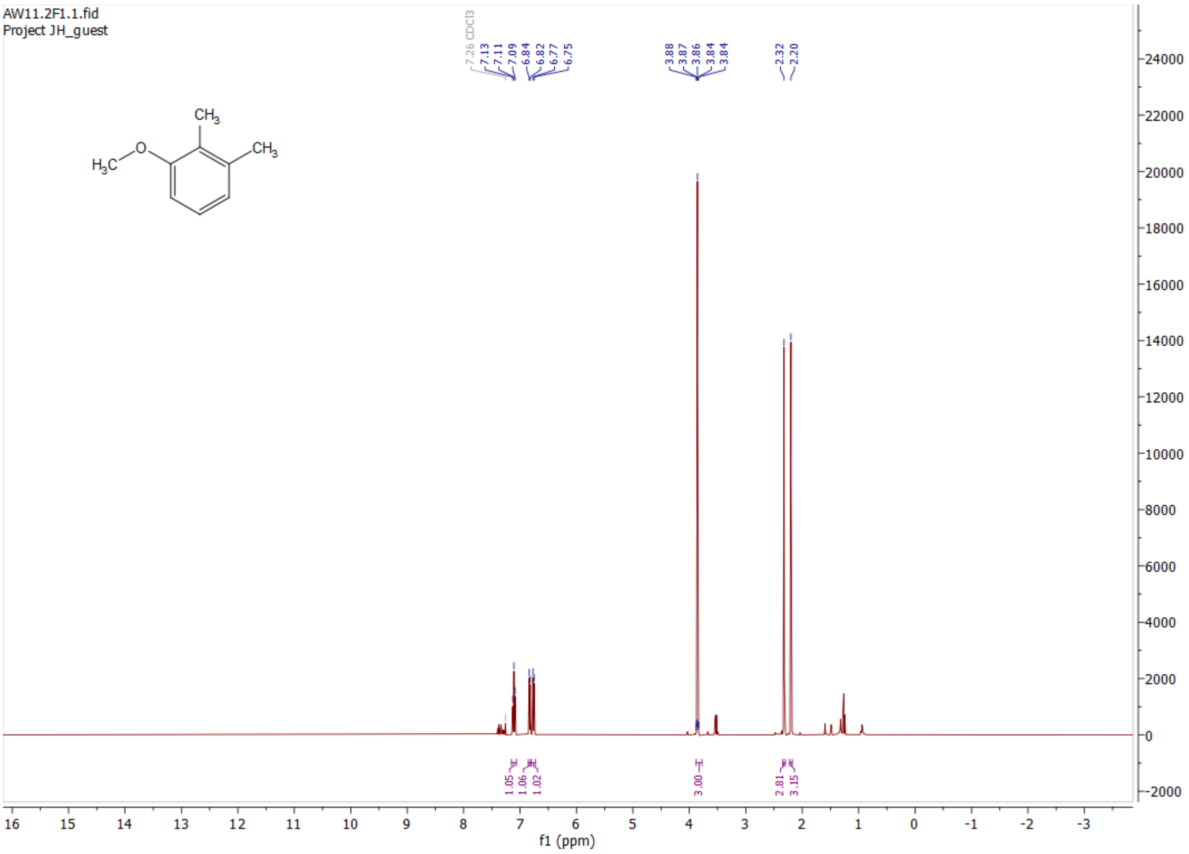
AW11.2F1.1.fid
Project JH_guest
7.26 CDCl3
7.13
7.11
7.09
6.84
6.82
6.77
6.75
3.88
3.87
3.86
3.84
3.84
2.32
2.20
CH3
H3C
O
CH3
1.05
1.06
1.02
3.00
2.81
3.15
f1 (ppm)

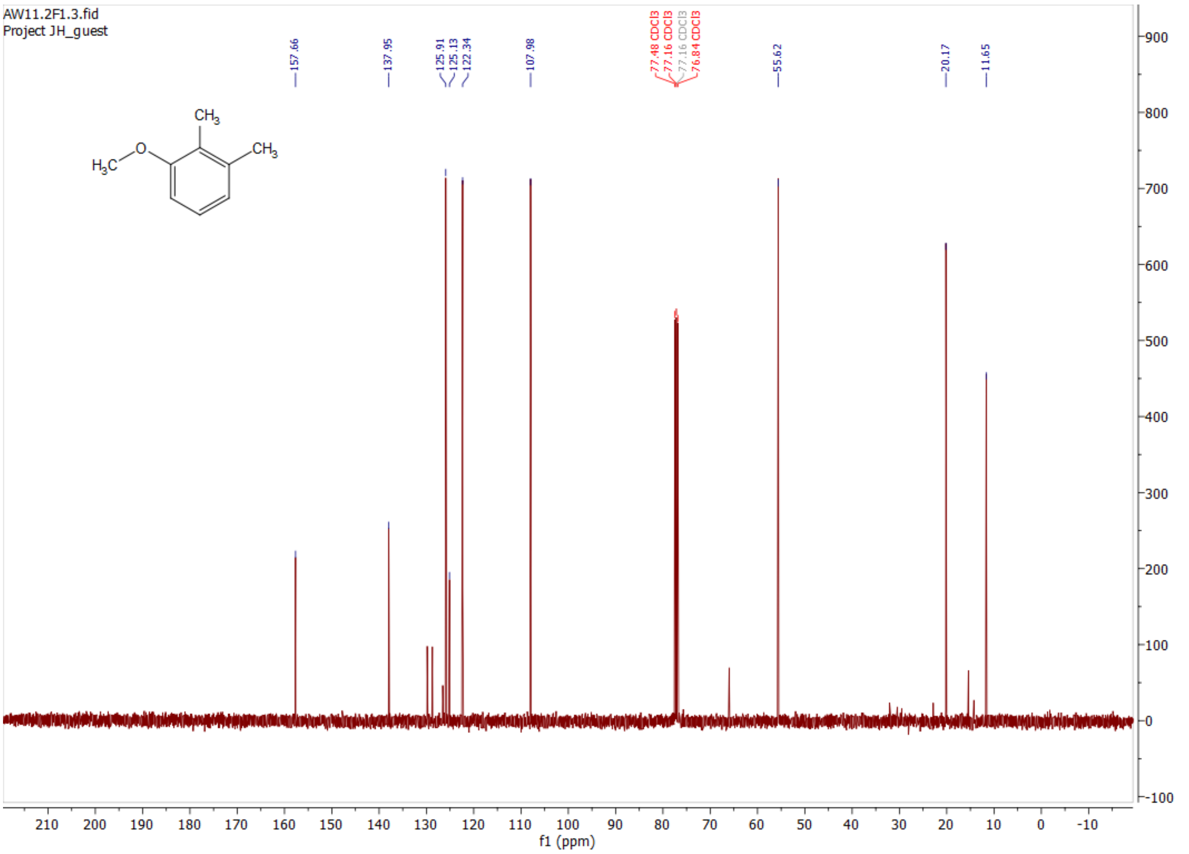
AW11.2F1.3.fid
Project JH_guest
157.66
137.95
125.91
125.13
122.34
107.98
77.48 CDCl3
77.16 CDCl3
77.16 CDCl3
76.84 CDCl3
55.62
20.17
11.65
CH3
H3C
O
CH3
f1 (ppm)

Data for 2o

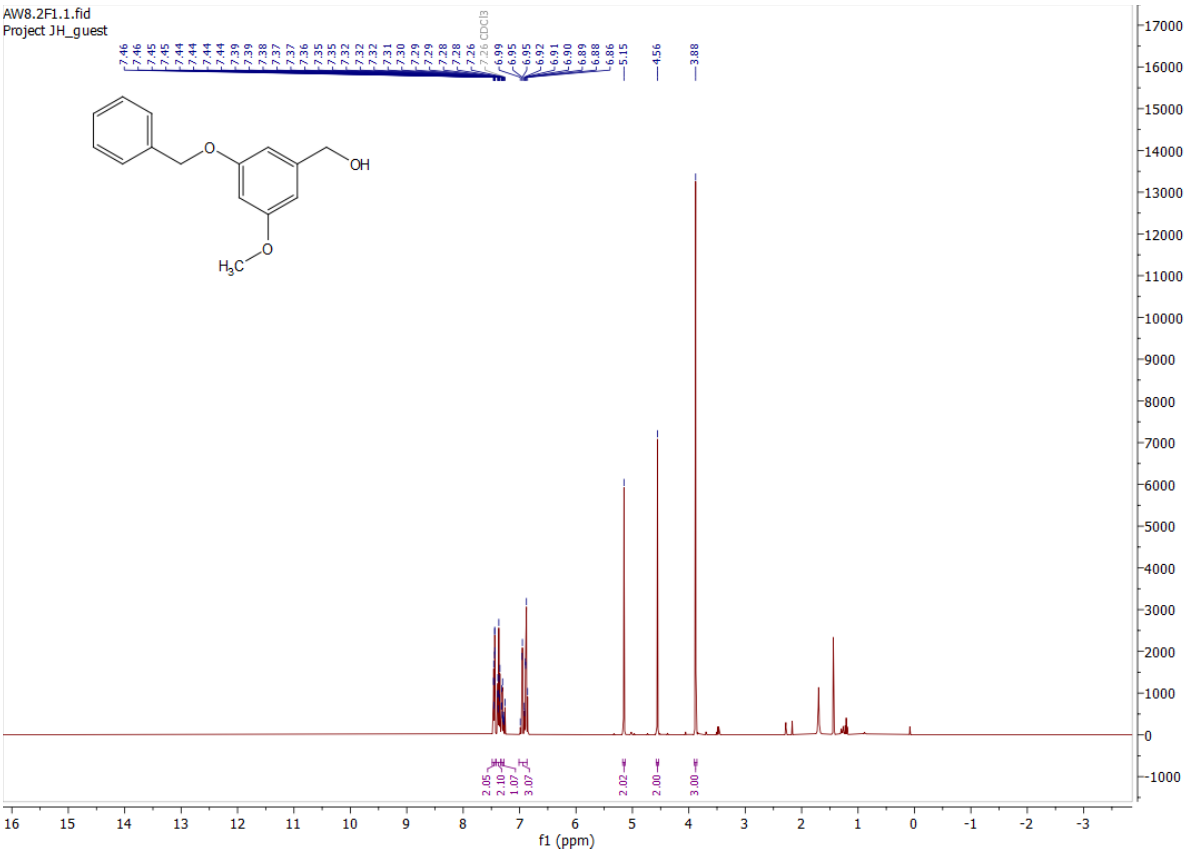

AW8.2F1.1.fid
Project JH_guest
7.46
7.46
7.45
7.45
7.44
7.44
7.44
7.44
7.39
7.39
7.38
7.37
7.37
7.36
7.35
7.35
7.32
7.32
7.32
7.31
7.30
7.29
7.29
7.28
7.28
7.26
7.26 CDCl3
6.99
6.95
6.95
6.92
6.91
6.90
6.89
6.88
6.86
5.15
4.56
3.88
OH
O
H3C
2.05
2.10
1.07
3.07
2.02
2.00
3.00
f1 (ppm)

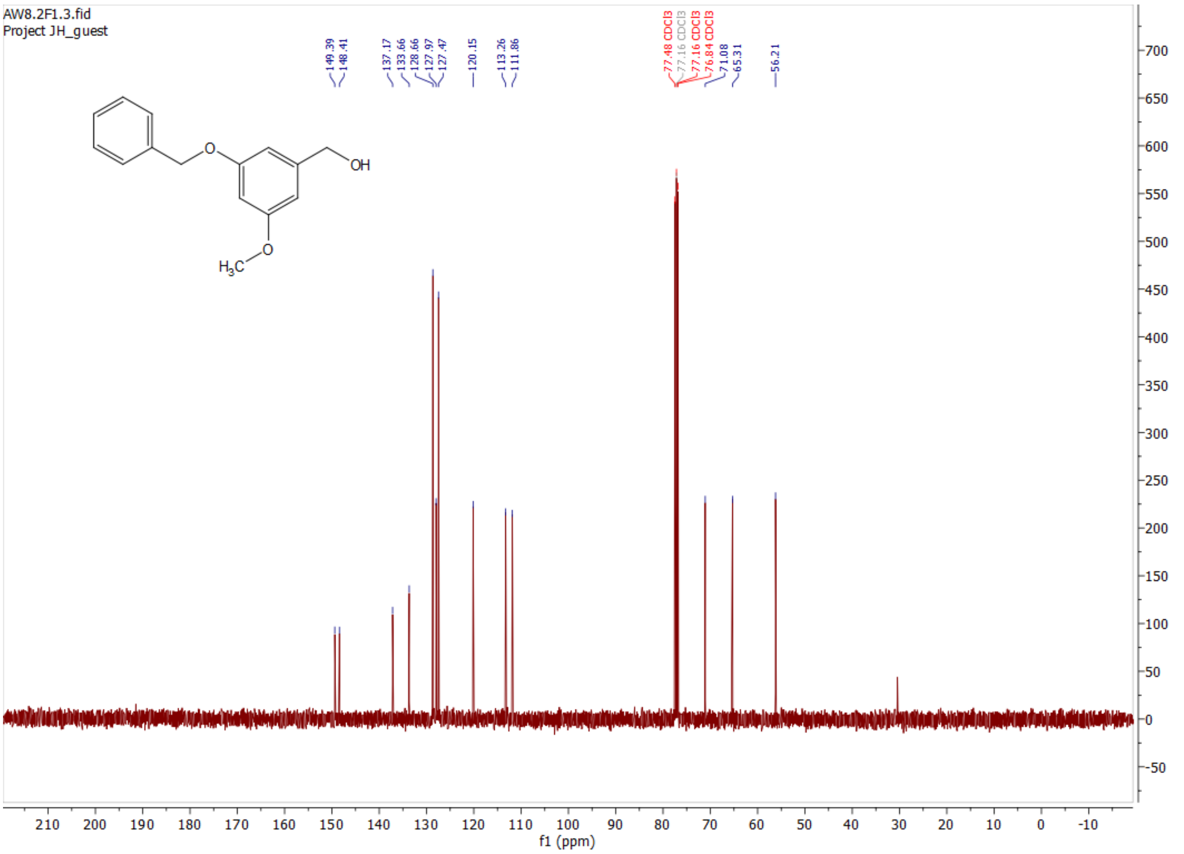

AW8.2F1.3.fid
Project JH_guest
149.39
148.41
137.17
133.66
128.66
127.97
127.47
120.15
113.26
111.86
77.48 CDCl3
77.16 CDCl3
77.16 CDCl3
76.84 CDCl3
71.08
65.31
56.21
OH
O
H3C
f1 (ppm)

Data for 2p

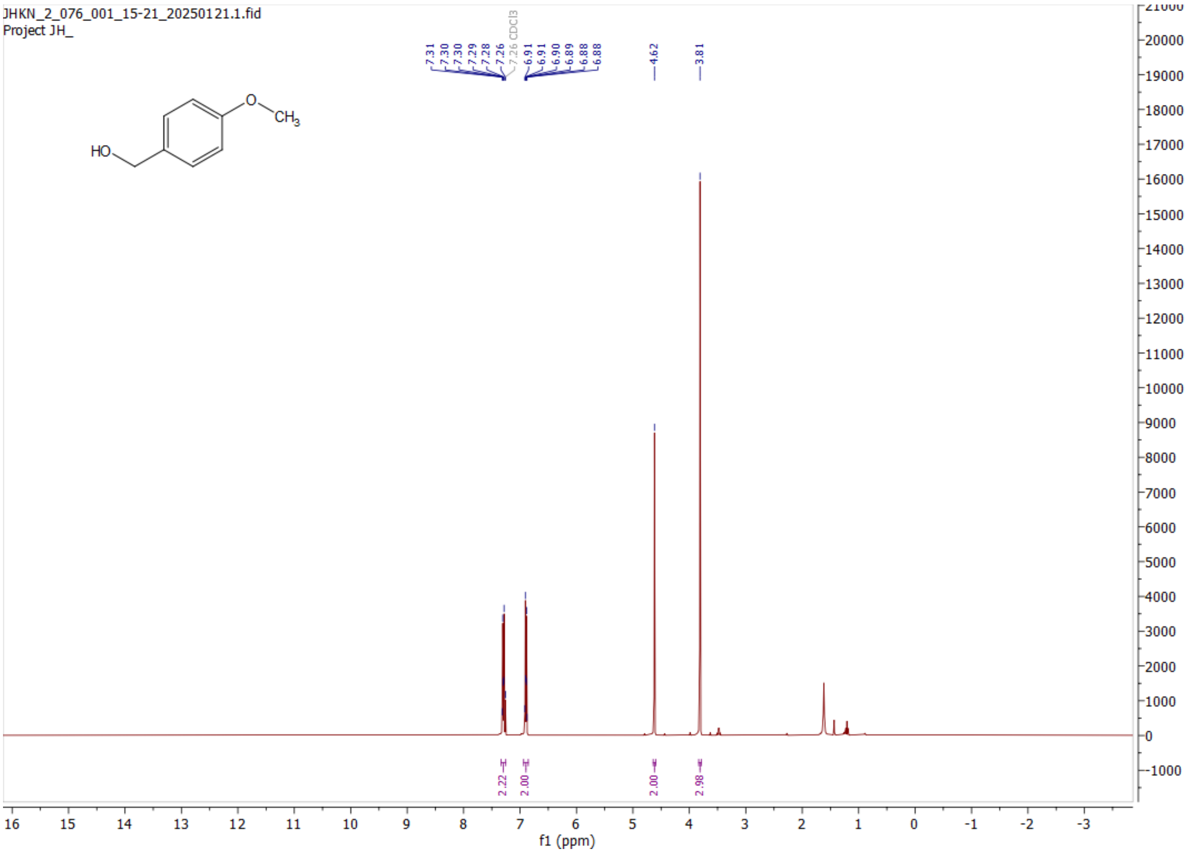
JHKN_2_076_001_15-21_20250121.1.fid
Project JH_
HO
O
CH3
7.31
7.30
7.30
7.29
7.28
7.26
7.26 CDCl3
6.91
6.91
6.90
6.89
6.88
6.88
4.62
3.81
2.22
2.00
2.00
2.98
f1 (ppm)

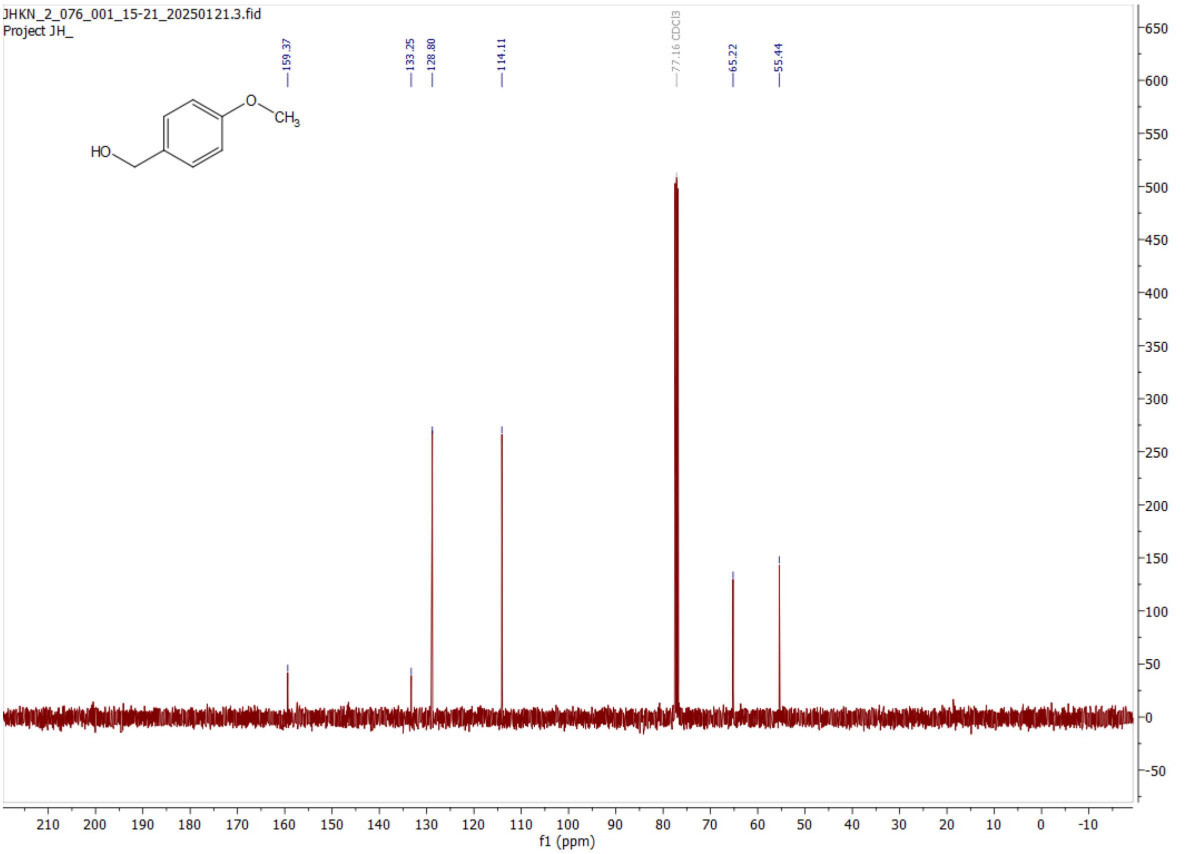
JHKN_2_076_001_15-21_20250121.3.fid
Project JH_
HO
O
CH3
159.37
133.25
128.80
114.11
77.16 CDCl3
65.22
55.44
f1 (ppm)

Data for 2q

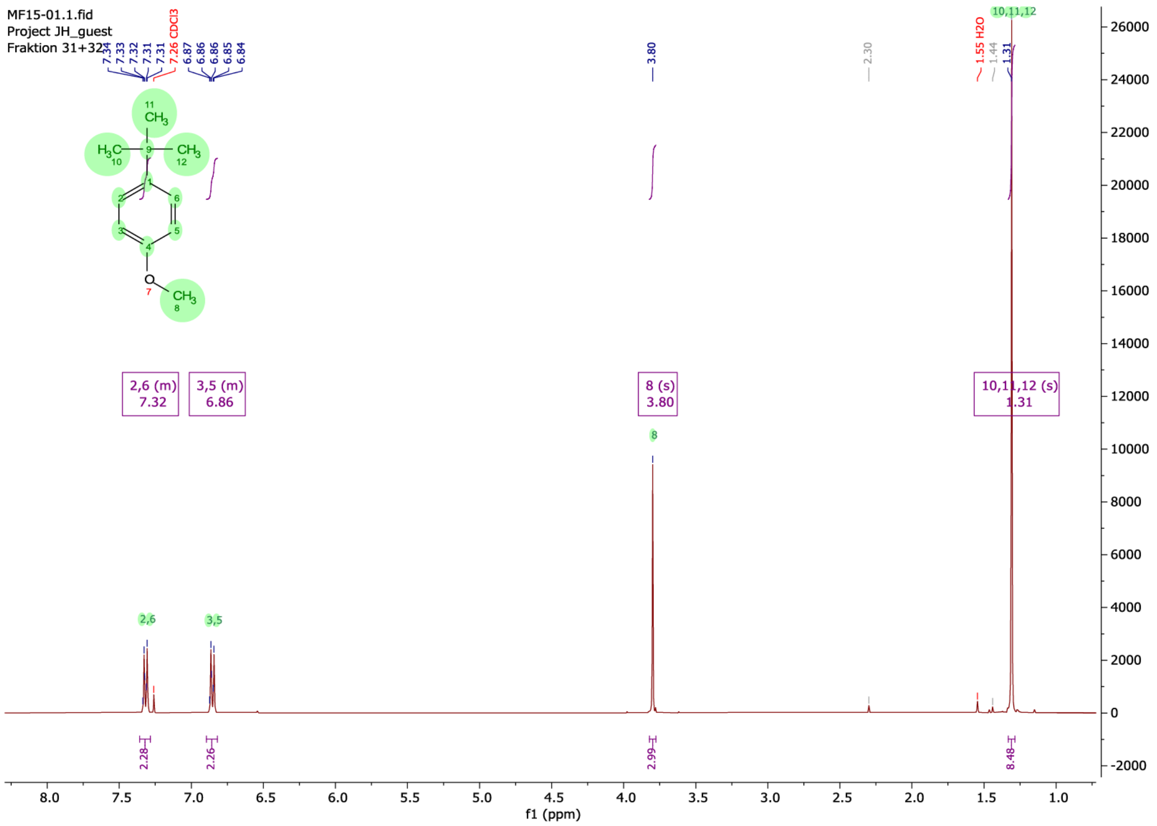

MF15-01.1.fid
Project JH_guest
Fraktion 31+32
7.34 7.33 7.32 7.31 7.31 7.26 CDCl3 6.87 6.86 6.86 6.85 6.84
3.80
2.30
1.55 H2O
1.44
1.31
2,6 (m) 7.32
3,5 (m) 6.86
8 (s) 3.80
10,11,12 (s) 1.31
2.28
2.26
2.99
8.48
f1 (ppm)

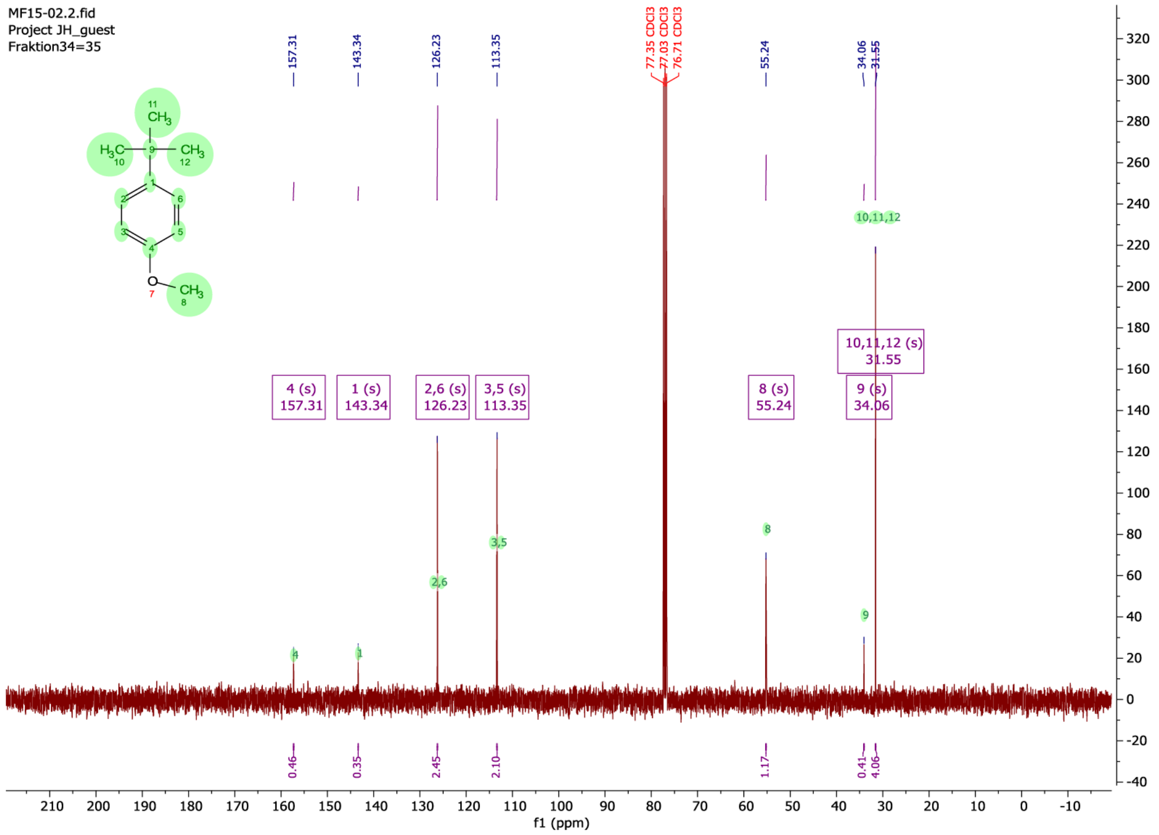

MF15-02.2.fid
Project JH_guest
Fraktion34=35
157.31
143.34
126.23
113.35
77.35 CDCl3
77.03 CDCl3
76.71 CDCl3
55.24
34.06
31.55
4 (s) 157.31
1 (s) 143.34
2,6 (s) 126.23
3,5 (s) 113.35
8 (s) 55.24
9 (s) 34.06
10,11,12 (s) 31.55
0.46
0.35
2.45
2.10
1.17
0.41
4.06
f1 (ppm)

Data for 2r

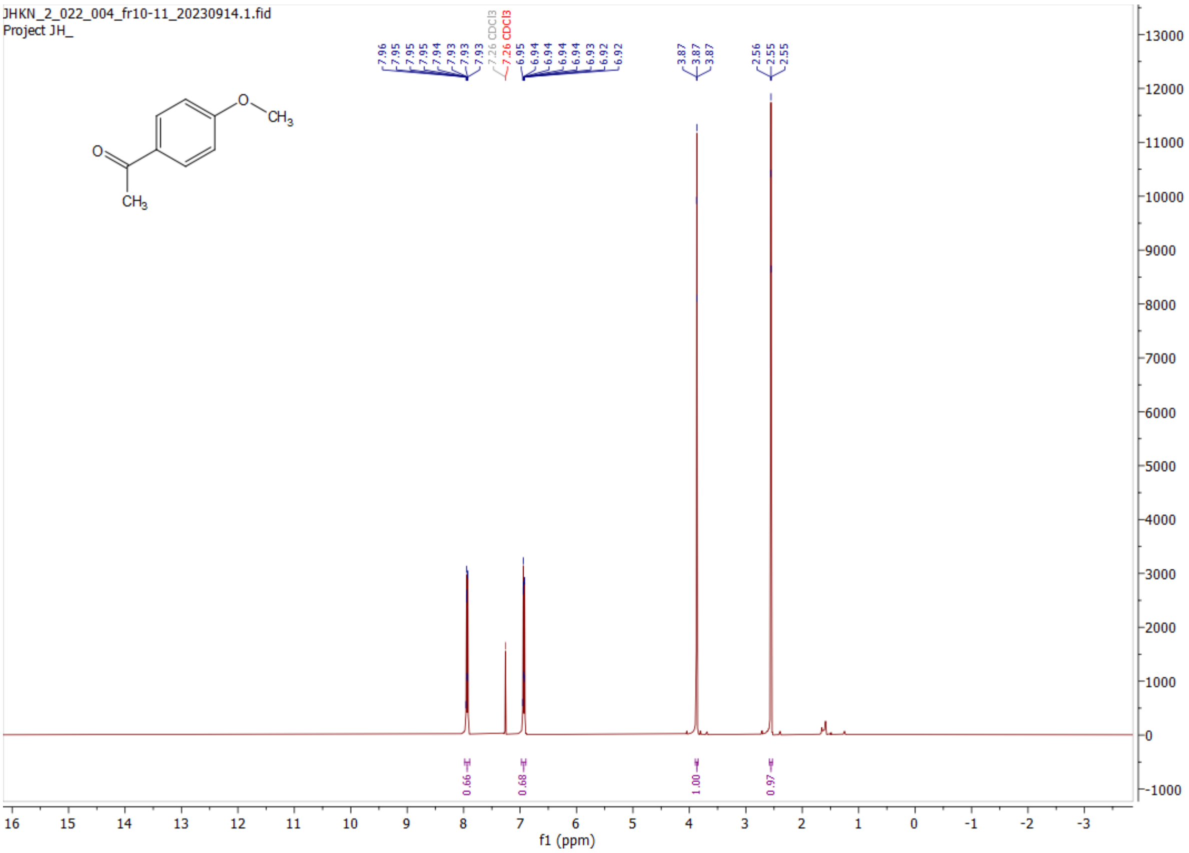
JHKN_2_022_004_fr10-11_20230914.1.fid
Project JH_
7.96
7.95
7.95
7.95
7.94
7.93
7.93
7.93
7.26 CDCl3
7.26 CDCl3
6.95
6.94
6.94
6.94
6.94
6.93
6.92
6.92
3.87
3.87
3.87
2.56
2.55
2.55
0.66
0.68
1.00
0.97
f1 (ppm)

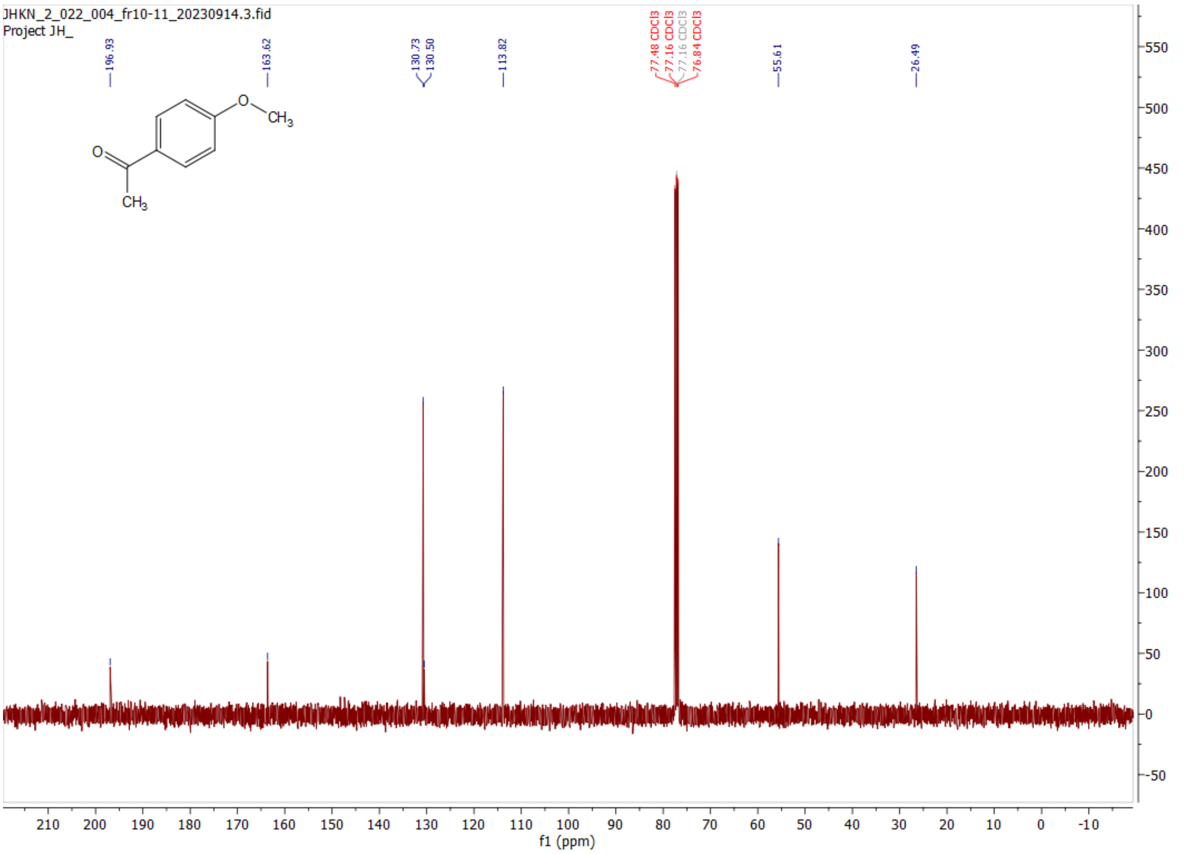
JHKN_2_022_004_fr10-11_20230914.3.fid
Project JH_
196.93
163.62
130.73
130.50
113.82
77.48 CDCl3
77.16 CDCl3
77.16 CDCl3
76.84 CDCl3
55.61
26.49
f1 (ppm)

Data for 2s

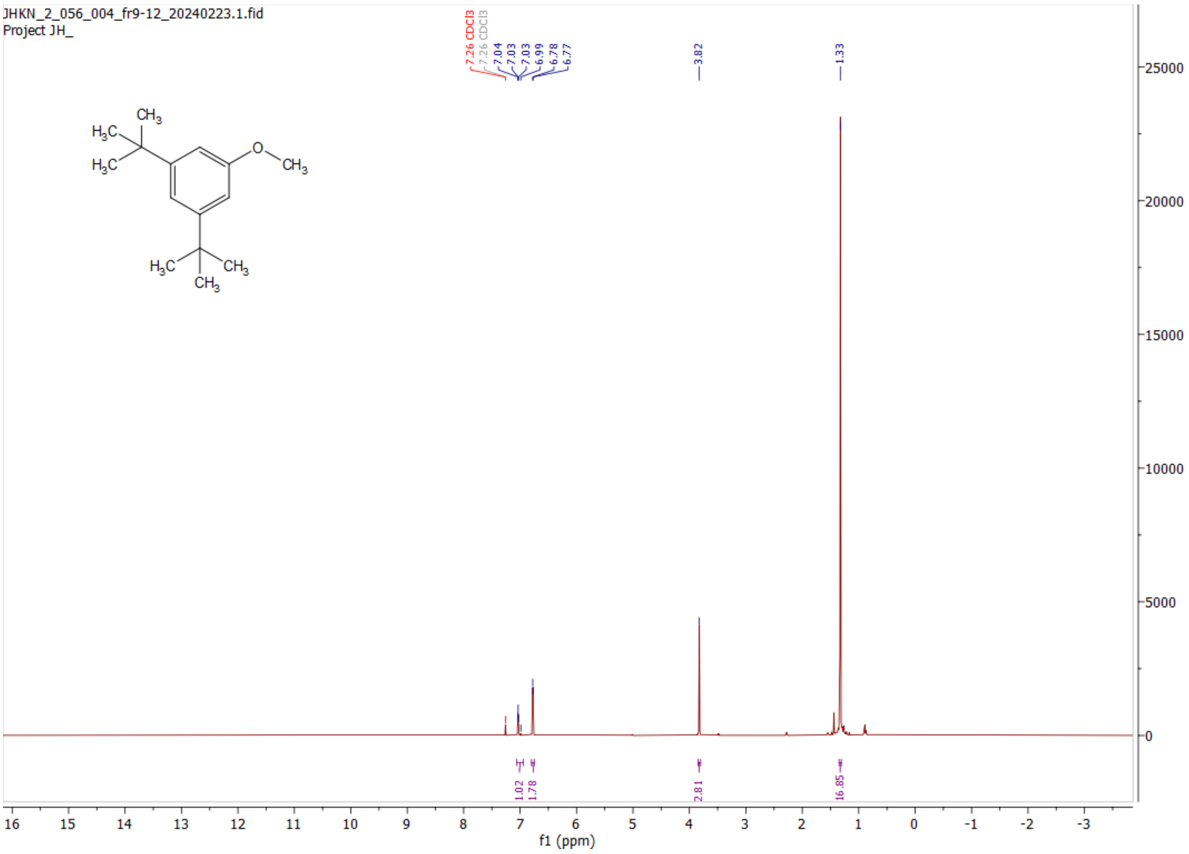
JHKN_2_056_004_fr9-12_20240223.1.fid
Project JH_
7.26 CDCl3
7.26 CDCl3
7.04
7.03
7.03
6.99
6.78
6.77
3.82
1.33
1.02
1.78
2.81
16.85
f1 (ppm)

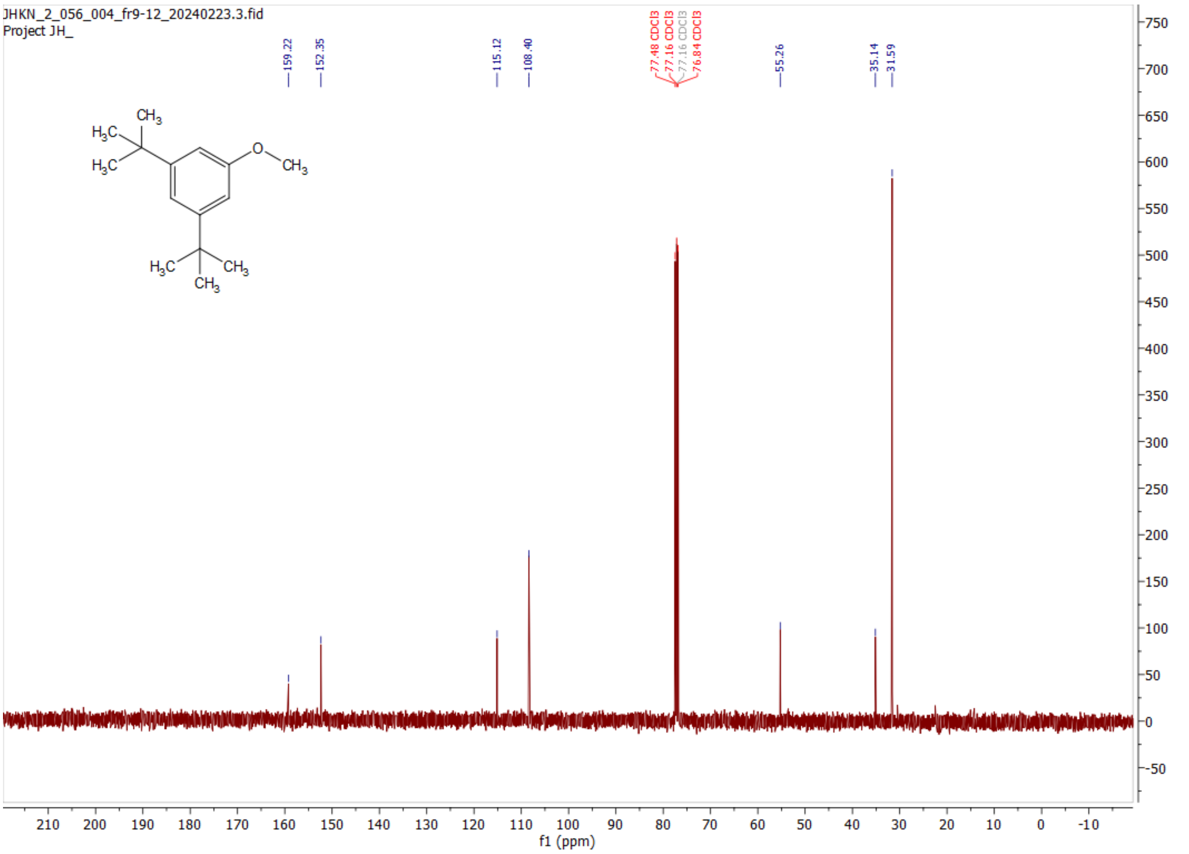
JHKN_2_056_004_fr9-12_20240223.3.fid
Project JH_
159.22
152.35
115.12
108.40
77.48 CDCl3
77.16 CDCl3
77.16 CDCl3
76.84 CDCl3
55.26
35.14
31.59
f1 (ppm)

Data for 2t

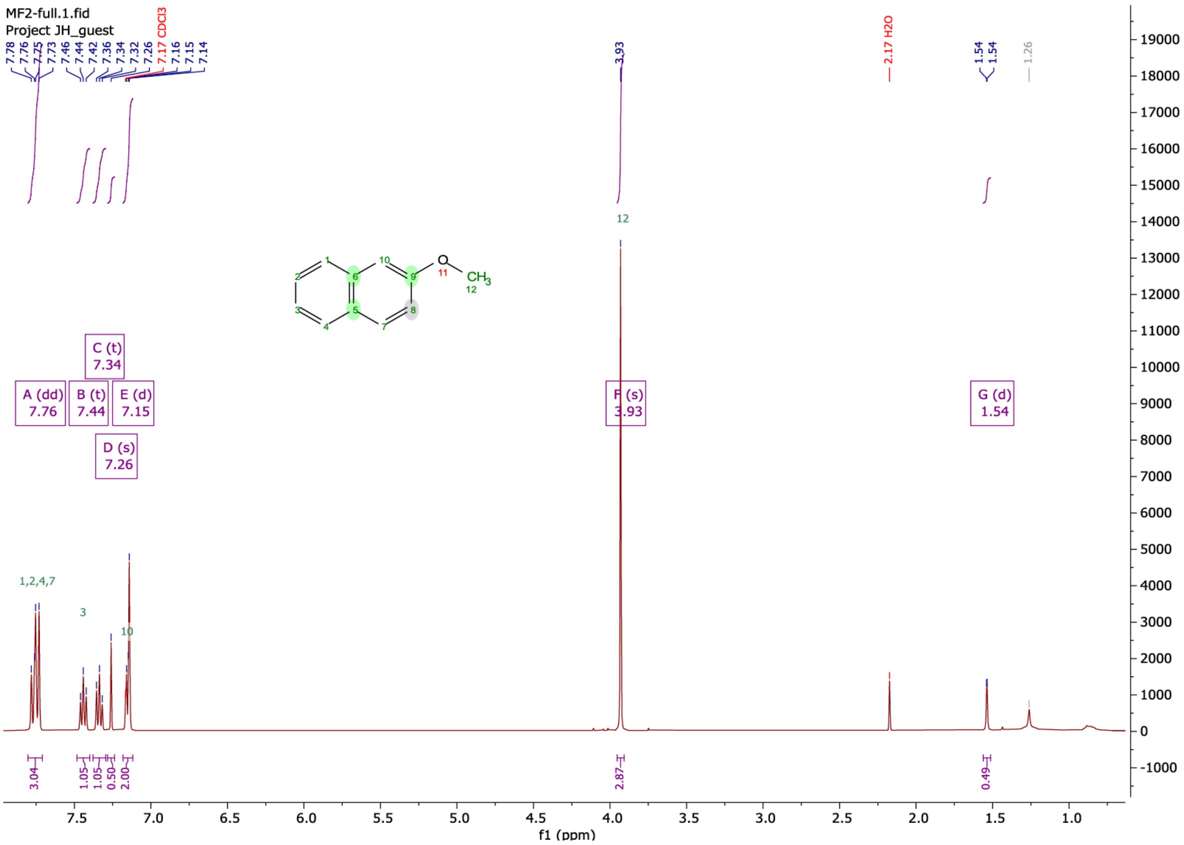
MF2-full.1.fid
Project JH_guest
A (dd) 7.76
B (t) 7.44
C (t) 7.34
D (s) 7.26
E (d) 7.15
F (s) 3.93
G (d) 1.54
2.17 H2O
7.17 CDCl3
f1 (ppm)

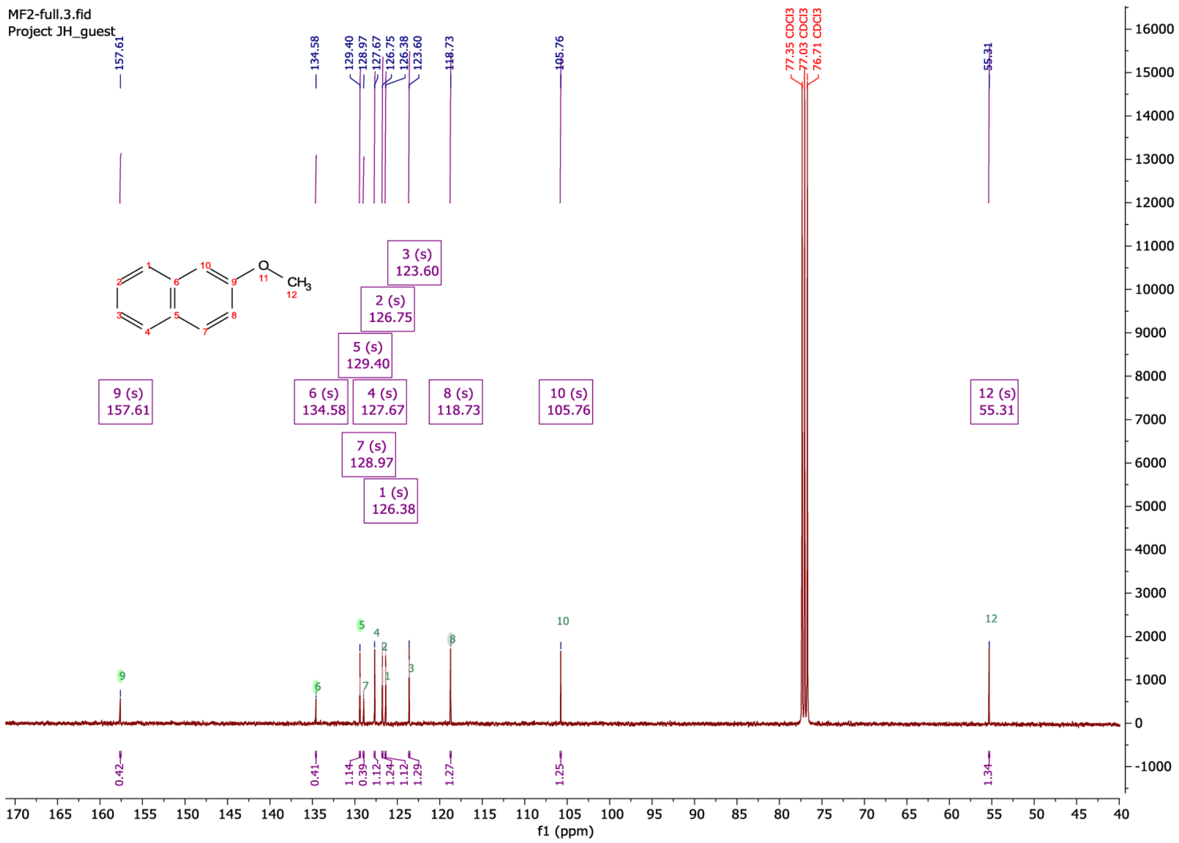
MF2-full.3.fid
Project JH_guest
9 (s) 157.61
6 (s) 134.58
4 (s) 127.67
5 (s) 129.40
2 (s) 126.75
3 (s) 123.60
7 (s) 128.97
1 (s) 126.38
8 (s) 118.73
10 (s) 105.76
12 (s) 55.31
77.35 CDCl3
77.03 CDCl3
76.71 CDCl3
f1 (ppm)

Data for 2u

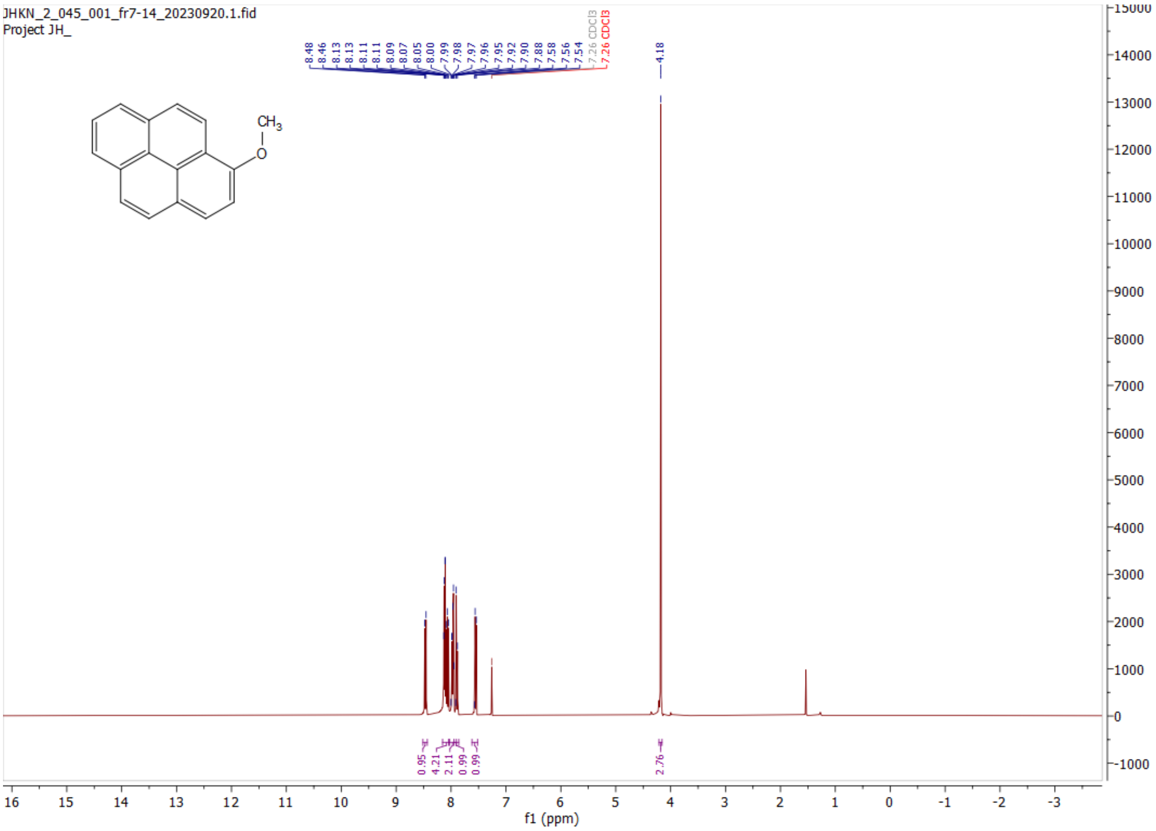
JHKN_2_045_001_fr7-14_20230920.1.fid
Project JH_
CH3
O
8.48
8.46
8.13
8.13
8.11
8.11
8.09
8.07
8.05
8.00
7.99
7.98
7.97
7.96
7.95
7.92
7.90
7.88
7.58
7.56
7.54
7.26 CDCl3
7.26 CDCl3
4.18
0.95
4.21
2.11
0.99
0.99
2.76
f1 (ppm)

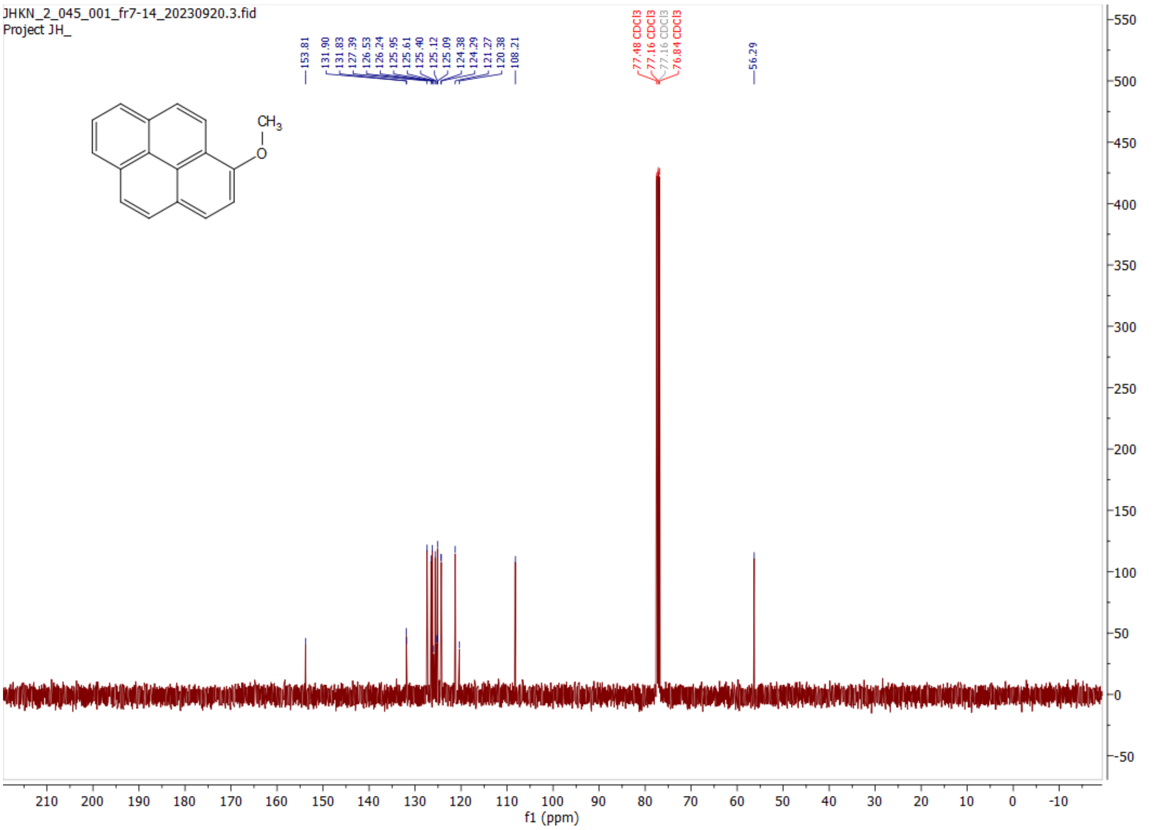
JHKN_2_045_001_fr7-14_20230920.3.fid
Project JH_
CH3
O
153.81
131.90
131.83
127.39
126.53
126.24
125.95
125.61
125.40
125.12
125.09
124.38
124.29
121.27
120.38
108.21
77.48 CDCl3
77.16 CDCl3
77.16 CDCl3
76.84 CDCl3
56.29
f1 (ppm)

Data for 2v

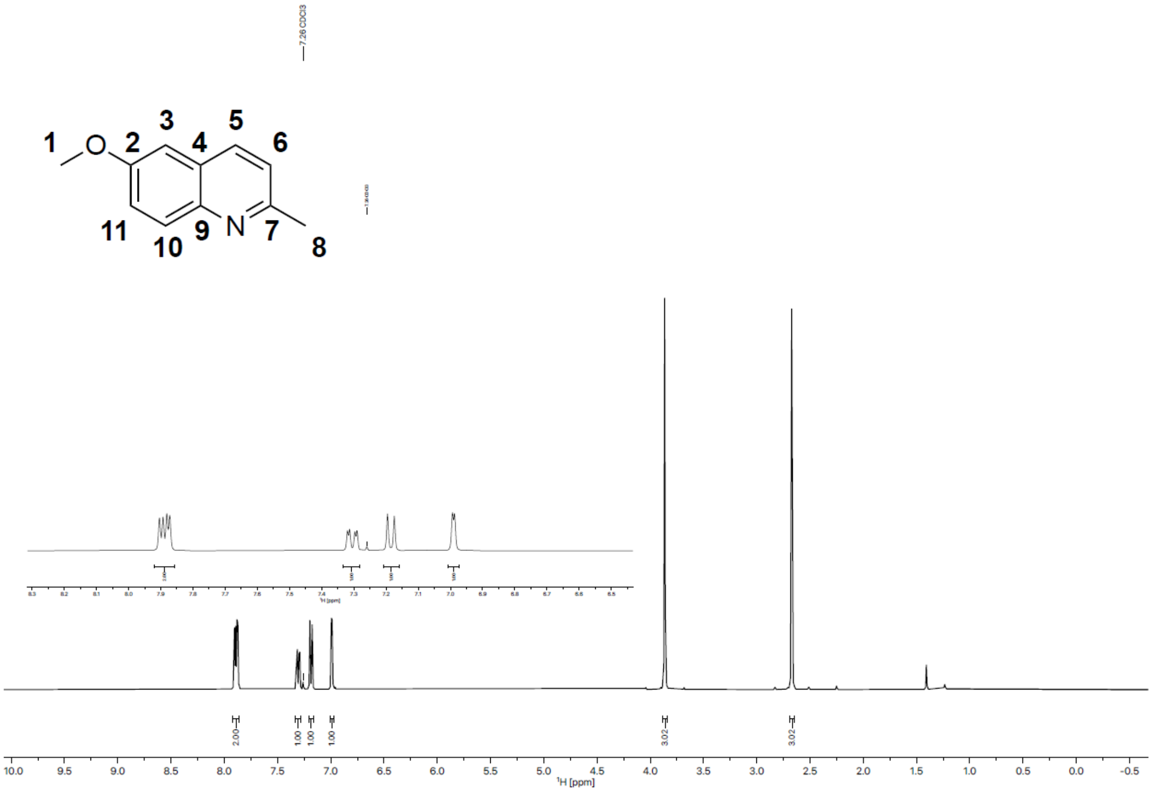
7.26 CDCl3
1
O
2
3
4
5
6
7
8
9
10
11
N
2.00
1.00
1.00
1.00
3.02
3.02
10.0 9.5 9.0 8.5 8.0 7.5 7.0 6.5 6.0 5.5 5.0 4.5 4.0 3.5 3.0 2.5 2.0 1.5 1.0 0.5 0.0 -0.5
1H [ppm]

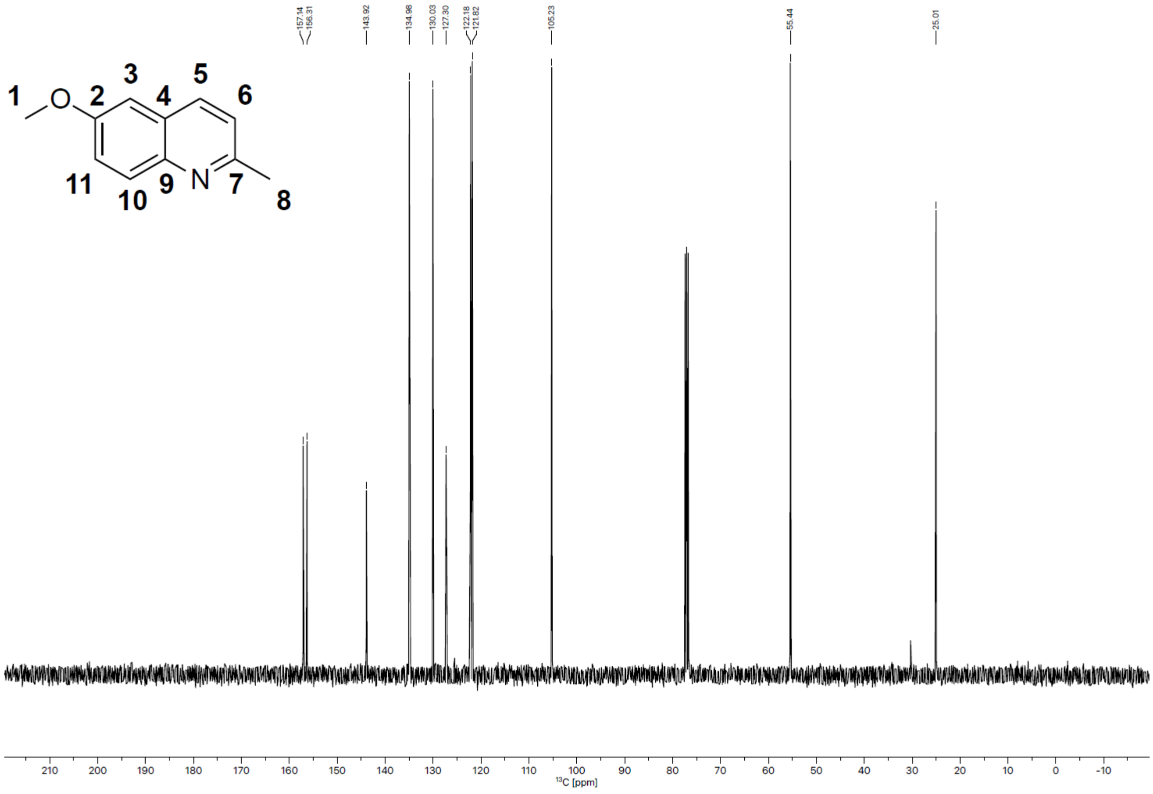
157.14
156.31
143.92
134.98
130.03
127.30
122.18
121.82
105.23
55.44
25.01
1
O
2
3
4
5
6
7
8
9
10
11
N
210 200 190 180 170 160 150 140 130 120 110 100 90 80 70 60 50 40 30 20 10 0 -10
13C [ppm]

Data for 2w

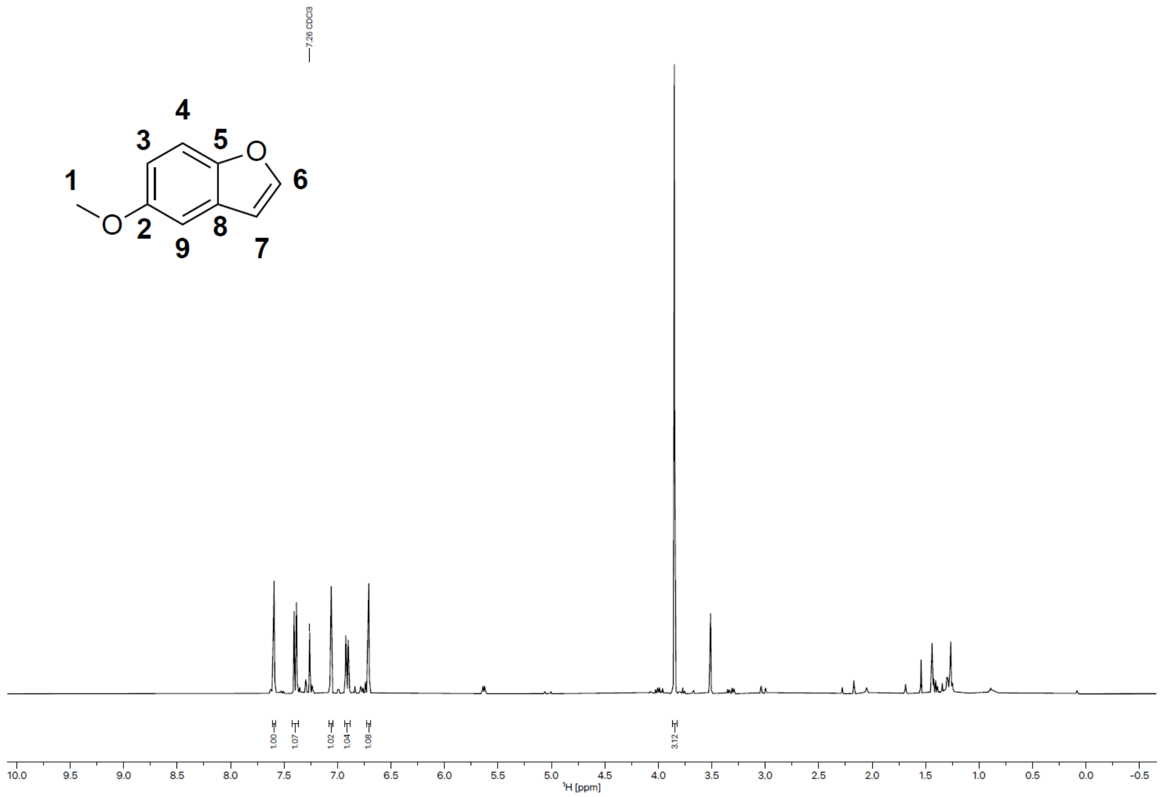
7.26 CDCl3
1
2
3
4
5
6
7
8
9
O
O
1.00
1.07
1.02
1.04
1.08
3.12
1H [ppm]

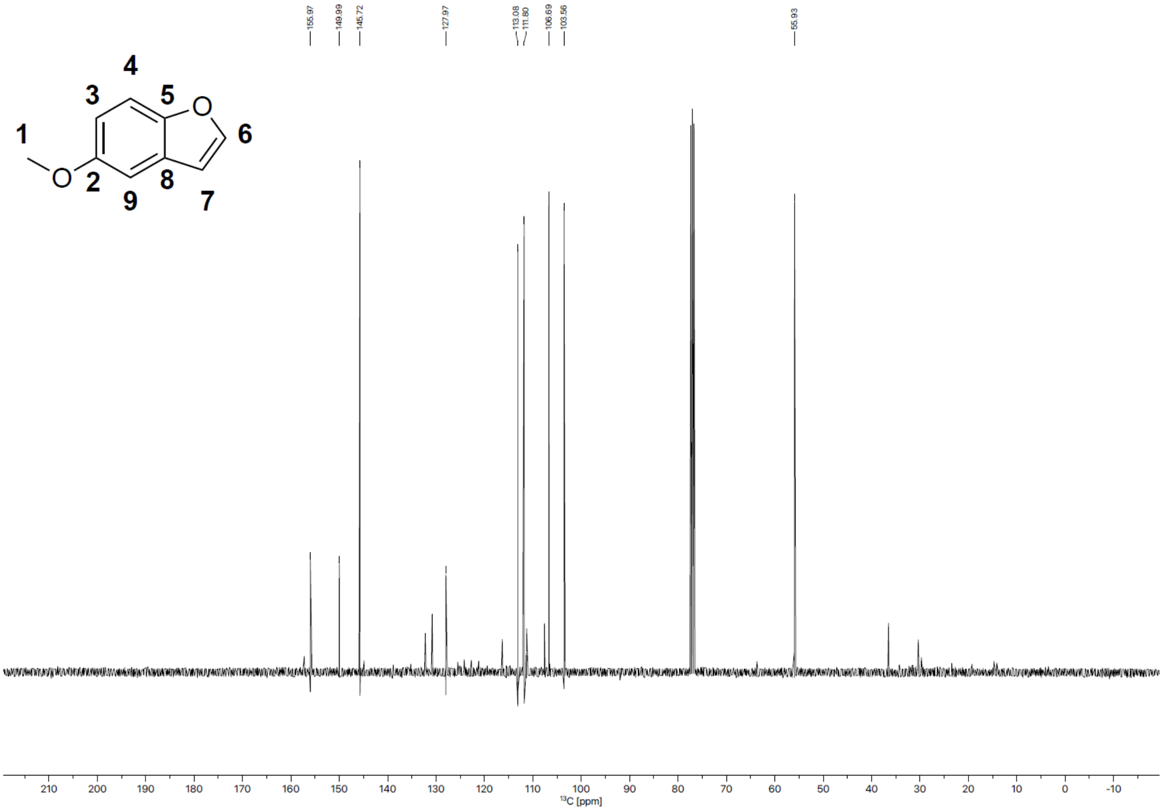
155.97
149.99
145.72
127.97
113.08
111.80
106.69
103.56
55.93
1
2
3
4
5
6
7
8
9
O
O
13C [ppm]

Data for 2x

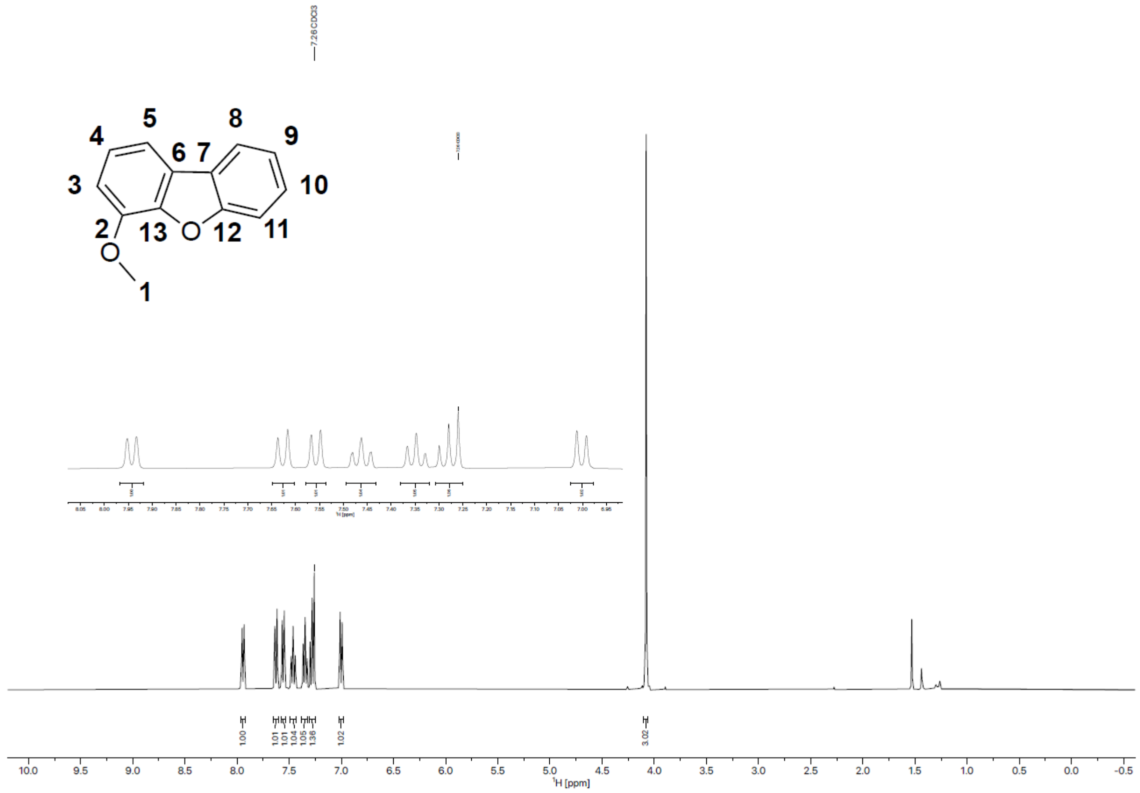

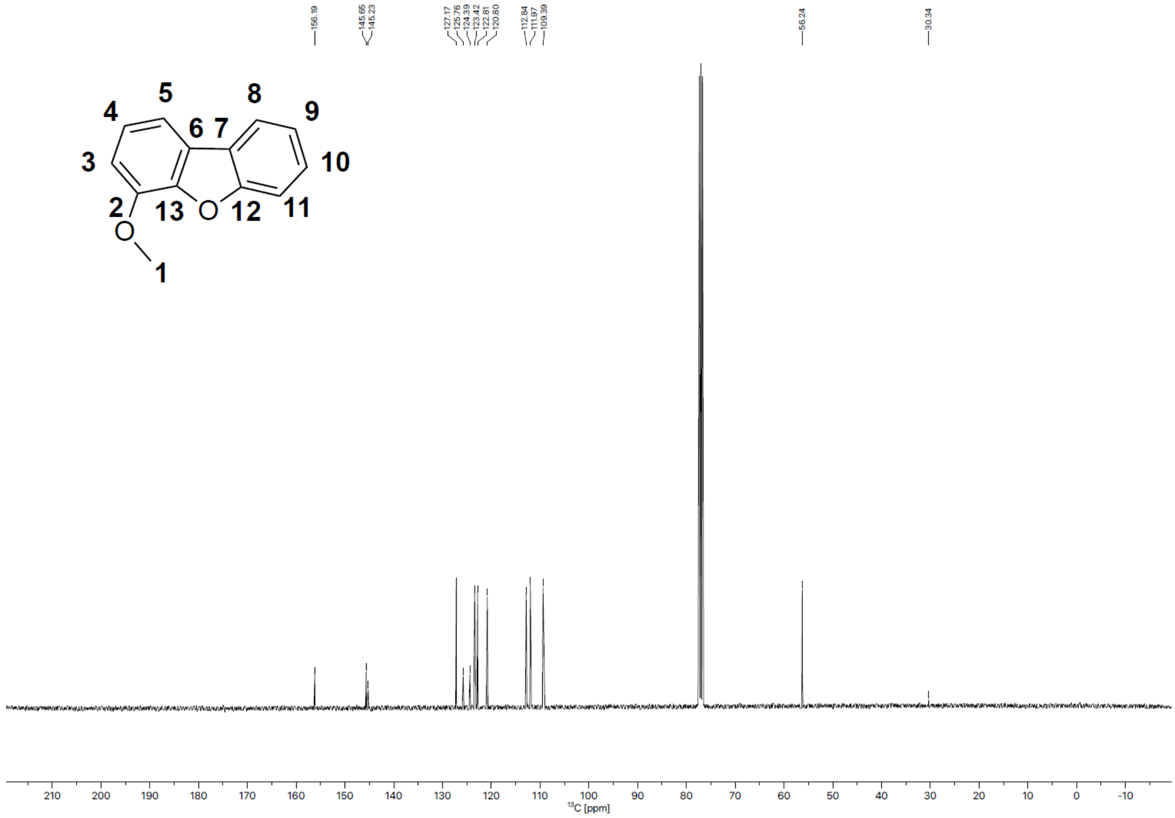

Crude NMR-data for 3 (^{13}C-labelled 2u)

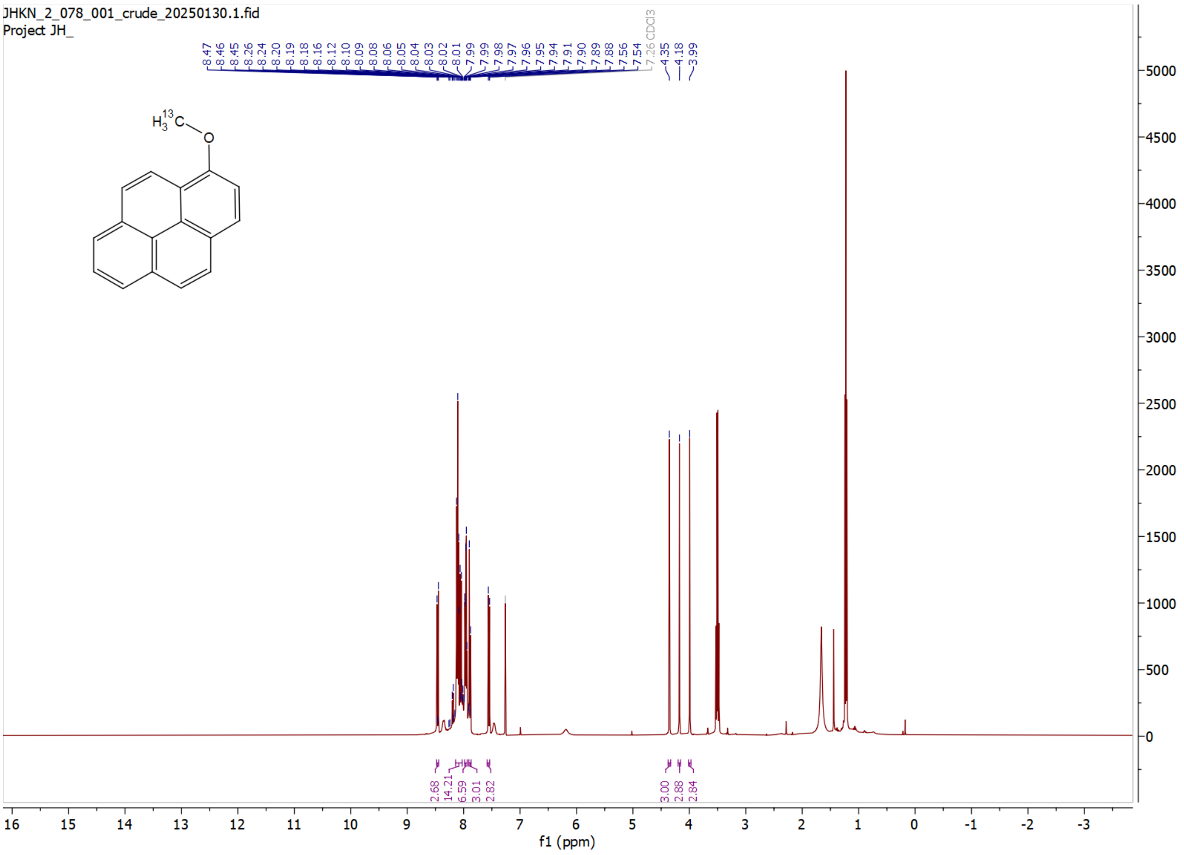

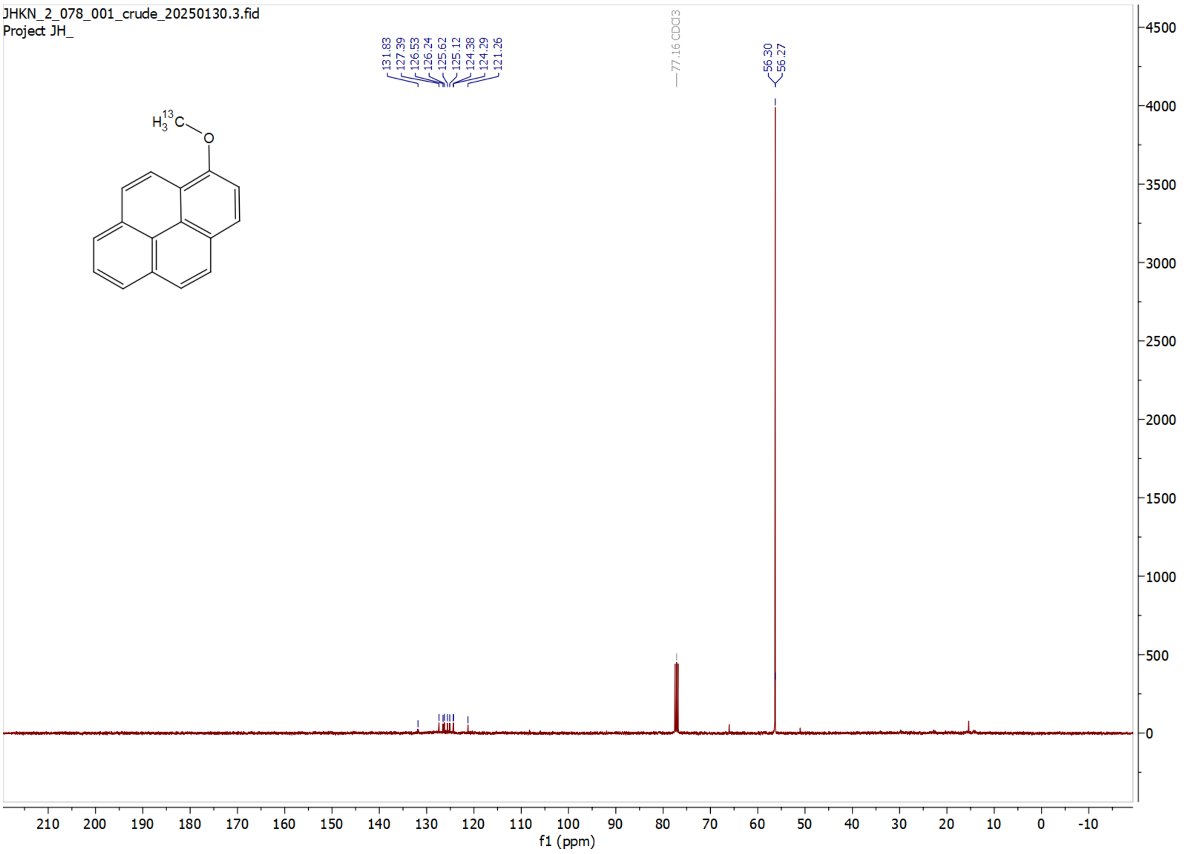

6. References

Asahara, K. K., Okita, T., Saito, A. N., Muto, K., Nakao, Y., & Yamaguchi, J. (2019). Pd-Catalyzed Denitrative Intramolecular C-H Arylation. *Organic Letters*, *21*(12), 4721-4724.

Bai, X. F., Ye, F., Zheng, L. S., Lai, G. Q., Xia, C. G., & Xu, L. W. (2012). Hydrosilane and bismuth-accelerated palladium catalyzed aerobic oxidative esterification of benzylic alcohols with air. *Chemical Communications*, *48*(68), 8592-8594.

Bhadra, S., Dzik, W. I., & Goossen, L. J. (2013). Synthesis of Aryl Ethers from Benzoates through Carboxylate-Directed C-H-Activating Alkoxylation with Concomitant Protodecarboxylation. *Angewandte Chemie-International Edition*, *52*(10), 2959-2962.

Cheung, C. W., & Buchwald, S. L. (2013). Mild and General Palladium-Catalyzed Synthesis of Methyl Aryl Ethers Enabled by the Use of a Palladacycle Precatalyst. *Organic Letters*, *15*(15), 3998-4001.

Collins, J. L. F., A. M.; Maloney, P. R.; Stewart, E. L.; Willson, T. M. (2004). *Substitued aminopropoxyaryl derivatives useful as agonists for lxr*. US-patent US2004072868A1.

Eskildsen, J., Christensen, T., Reenberg, T., Larsen, U., & Christensen, J. B. (2000). An improved synthesis of 2,3-bis(bromomethyl)-1,4-dimethoxybenzene. *Organic Preparations and Procedures International*, *32*(4), 398-400.

Fan, H. L., Sun, H. B., & Peng, X. H. (2018). Substituents Have a Large Effect on Photochemical Generation of Benzyl Cations and DNA Cross-Linking. *Chemistry-a European Journal*, *24*(30), 7671-7682.

Haydl, A. M., & Hartwig, J. F. (2019). Palladium-Catalyzed Methylation of Aryl, Heteroaryl, and Vinyl Boronate Esters. *Organic Letters*, *21*(5), 1337-1341.

He, K. H., Tan, F. F., Zhou, C. Z., Zhou, G. J., Yang, X. L., & Li, Y. (2017). Acceptorless Dehydrogenation of N-Heterocycles by Merging Visible-Light Photoredox Catalysis and Cobalt Catalysis. *Angewandte Chemie-International Edition*, *56*(11), 3080-3084.

Huang, C. H., Liang, T., Harada, S., Lee, E., & Ritter, T. (2011). Silver-Mediated Trifluoromethoxylation of Aryl Stannanes and Arylboronic Acids. *Journal of the American Chemical Society*, *133*(34), 13308-13310.

Kawabata, T., & Matsubara, H. (2023). Safe and facile evolution of diazomethane using the phase-vanishing method. *Tetrahedron Letters*, *123*.

Liang, Y. J., Lin, F. G. R., Adeli, Y., Jin, R., & Jiao, N. (2019). Efficient Electrocatalysis for the Preparation of (Hetero)aryl Chlorides and Vinyl Chloride with 1,2-Dichloroethane. *Angewandte Chemie-International Edition*, *58*(14), 4566-4570.

Meng, Q. Y., Wang, S., & König, B. (2017). Carboxylation of Aromatic and Aliphatic Bromides and Triflates with CO by Dual Visible-Light-Nickel Catalysis. *Angewandte Chemie-International Edition*, *56*(43), 13426-13430.

Nawrat, C. C., Palmer, L. I., Blake, A. J., & Moody, C. J. (2013). Two Approaches to the Aromatic Core of the Aminonaphthoquinone Antibiotics. *Journal of Organic Chemistry*, *78*(11), 5587-5603.

Panda, S., Nanda, A., Behera, R. R., Ghosh, R., & Bagh, B. (2023). Cobalt catalyzed chemoselective reduction of nitroarenes: hydrosilylation under thermal and photochemical reaction conditions. *Chemical Communications*, *59*(30), 4527-4530.

Shen, Z. J., Wei, J. N., Hu, X., Gu, Z. M., Shao, Q. Q., Chen, X., Wang, M. M., & Zhang, D. L. (2025). Photoinduced ligand-to-copper charge transfer for decarboxylative hydrogenation of aromatic carboxylic acids. *Tetrahedron*, *175*.

Shi, L., Liu, Y. Y., Liu, Q. F., Wei, B., & Zhang, G. S. (2012). Selective reduction of aldehydes and ketones to alcohols with ammonia borane in neat water. *Green Chemistry*, *14*(5), 1372-1375.

Sum, T. J., Sum, T. H., Galloway, W. R. J. D., Twigg, D. G., Ciardiello, J. J., & Spring, D. R. (2018). Synthesis of structurally diverse biflavonoids. *Tetrahedron*, *74*(38), 5089-5101.

Sun, R., Qin, Y., & Nocera, D. G. (2020). General Paradigm in Photoredox Nickel-Catalyzed Cross-Coupling Allows for Light-Free Access to Reactivity. *Angewandte Chemie-International Edition*, *59*(24), 9527-9533.

Tang, Y. F., Huang, Y., Lu, L. X., Wang, C., Sun, T. M., Zhu, J. L., Zhu, G. H., Pan, J. Y., Jin, Y. L., Liu, A. L., & Wang, M. (2018). Synthesis of a new pyrene-devived fluorescent probe for the detection of Zn. *Tetrahedron Letters*, *59*(44), 3916-3922.

Wethman, R., Derosa, J., Tran, V., Kang, T., Apolinar, O., Abraham, A., Kleinmans, R., Wisniewski, S. R., Coombs, J. R., & Engle, K. M. (2021). An Under-Appreciated Source of Reproducibility Issues in Cross-Coupling: Solid-State Decomposition of Primary Sodium Alkoxides in Air. *ACS Catalysis*, *11*(2), 502-508.

Xu, N., Peng, X. Q., Luo, C., Huang, L., Wang, C. D., Chen, Z., & Li, J. J. (2023). Light-Promoted Chlorine-Radical-Mediated Oxidation of Benzylic C(sp)-H Bonds utilizing Air as Oxidant. *Advanced Synthesis & Catalysis*, *365*(2), 142-147.

Zhang, G. F., Wang, Y., Wen, X., Ding, C. R., & Li, Y. (2012). Dual-functional click-triazole: a metal chelator and immobilization linker for the construction of a heterogeneous palladium catalyst and its application for the aerobic oxidation of alcohols. *Chemical Communications*, *48*(24), 2979-2981.

Zhou, J., Jiang, B. Y., Zhao, Z. Y., & Shibata, N. (2022). Etherification of Fluoroarenes with Alkoxyboronic Acid Pinacol Esters via C-F Bond Cleavage. *Organic Letters*, *24*(28), 5084-5089.
